# Livestock landscapes as ecological filters: Effects of the tree cover gradient on the taxonomic and functional diversity of granivorous birds in the Colombian Amazon

**DOI:** 10.1371/journal.pone.0345283

**Published:** 2026-03-20

**Authors:** Jenniffer Tatiana Díaz-Cháux, Alexander Velasquez-Valencia, Alejandro Navarro-Morales, Fernando Casanoves

**Affiliations:** 1 Universidad de la Amazonia, Centro de Investigación de la Biodiversidad Andino Amazónica, Grupo de Investigación Fauna Silvestre, Facultad de Ciencias Básicas, Programa de Biología, Florencia, Caquetá, Colombia; 2 Universidad de la Amazonia, Facultad de Ciencias Agropecuarias, Programa de Doctorado en Ciencias Naturales y Desarrollo Sustentable, Florencia, Caquetá, Colombia; 3 Universidad de la Amazonia, Facultad de Ingeniería, Programa de Maestría en Bioestadística, Florencia, Caquetá, Colombia; 4 CATIE – Centro Agronómico Tropical de Investigación y Enseñanza, Turrialba, Costa Rica; Charles University: Univerzita Karlova, CZECHIA

## Abstract

Land-use intensification in cattle-ranching landscapes of the Colombian Amazon generates continuous gradients of tree cover that modify the structure of productive mosaics and may function as environmental filters. The effects of this gradient may vary depending on the biodiversity component and the scale of analysis considered. We evaluated how tree cover is associated with the abundance, taxonomic richness, and functional diversity of the granivorous bird guild in cattle-ranching landscapes of the Colombian Amazon. A total of 100 quadrats were sampled along the continuous tree cover gradient. Abundance and local richness were analyzed using generalized linear mixed models with a negative binomial distribution. Taxonomic diversity pooled by cover type was estimated using Hill numbers with explicit control of sampling completeness. Functional structure was assessed using multidimensional indices (FRic, FEve, FDiv, FDis, and RaoQ) and multivariate trait-based analyses (RLQ and fourth-corner tests). Tree cover had a significant effect on local abundance, whereas quadrat-level richness showed only a marginal response. Taxonomic diversity was higher in structurally more heterogeneous covers, consistent with greater spatial turnover. At the functional level, FDis and RaoQ varied among covers, whereas FRic and FEve did not differ significantly. RLQ analysis identified significant covariation between proportions of tree cover and traits associated with body size and bill and tarsus morphology in granivorous birds. Overall, the results describe a partial and scale-dependent reorganization of the granivorous assemblage along the structural gradient. Although consistent with theoretical expectations of environmental filtering, these patterns should be interpreted as observational associations rather than causal evidence or direct evaluations of ecosystem functions.

## Introduction

The Colombian Amazon constitutes one of the most globally significant socioecological systems due to its high biodiversity and the magnitude of ongoing land-use transformation processes. In recent decades, more than two million hectares of natural forest cover have been lost. The expansion of the agricultural frontier, land grabbing, and the increase in areas devoted to illicit crops [1–3] are the primary drivers of this deforestation. According to the Instituto de Hidrología, Meteorología y Estudios Ambientales de Colombia (IDEAM) [2], between 2018 and 2023 national deforestation was concentrated in the Amazon region, with an average annual loss of 95,000 ha of forest. In this context, the northwestern Colombian Amazon, where the department of Caquetá is located, represents the principal hotspot of forest cover loss and degradation, accounting for more than 20% of annual Amazonian deforestation during the same period [1,2]. In Caquetá, extensive cattle ranching constitutes one of the dominant land uses in transformed landscapes [4,5]. This activity has become the primary regional productive sector, with an inventory exceeding three million head of cattle, despite the low natural suitability of Amazonian soils, which require an average of 1.4 ha of pasture per animal to sustain production [4–6]. Between 2019 and 2023, livestock systems occupied 45% of the departmental agricultural area [3].

At the Amazonian scale, regional assessments consistently identify cattle ranching as one of the principal lands uses historically associated with deforestation and forest fragmentation, although its relative contribution varies spatially and temporally [7–11]. The spatial configuration of Amazonian cattle-ranching landscapes results from the historical interaction between land-use change and local physiography [12–14]. These processes have generated structural mosaics characterized by a continuous tree cover gradient, including open areas dominated by pastures, semi-open areas with scattered trees and early secondary regeneration, and semi-closed areas with greater canopy continuity [15,16]. These configurations do not represent discrete habitat types or independent production units, but rather dynamic structural states derived from clearing, pasture management, natural succession, and partial tree retention within livestock systems [17,18]. Within this framework, tree cover functions as an integrative gradient of the indirect effects of cattle ranching on landscape structure [19], modulating resource availability, refuge provision, and functional connectivity for wildlife. The figures presented above are provided strictly for contextual purposes, acknowledging their interannual and regional variability, and are used solely to frame the relevance of the tree cover gradient evaluated in this study, without implying direct causal relationships between deforestation, livestock activity, and the ecological patterns analyzed.

From the perspective of classical ecology, the relationship between biological diversity and habitat structure has been interpreted through foundational principles such as environmental heterogeneity, carrying capacity, and the ecological trade-offs faced by species along environmental gradients [20–24]. Habitat heterogeneity theory proposes that moderate increases in structural complexity expand the number of available niches and promote species coexistence [23–25]. However, it also recognizes nonlinear responses, whereby extreme simplification or fragmentation reduces the landscape’s capacity to sustain viable populations, leading to declines in richness and abundance within certain functional guilds [21,25,26]. In productive landscapes, these patterns emerge from trade-offs among resource availability, structural connectivity, and ecological costs associated with differential use of open versus tree-covered environments.

Contemporary functional ecology approaches have integrated these principles within an explicitly mechanistic framework, establishing that functional traits predictably determine both species’ responses to disturbance gradients and their potential contributions to ecosystem functioning [27–30]. Under environmental filtering theory, landscape configuration operates as a set of hierarchical filters that select specific trait combinations, constraining species persistence in transformed environments through limitations imposed by habitat structure and resource availability [31–33]. In birds, these filters primarily act on traits related to body size, mobility, and trophic specialization, which modulate physiological tolerance to environmental stress, movement capacity across open matrices, and differential access to resources [34].

In fragmented and productive landscapes, the occupation of forest remnants by bird species depends critically on their ability to traverse open areas [28,29]. These processes are closely linked to mobility and body size, as well as landscape attributes such as fragment size, connectivity, and conservation status [35]. Structural habitat simplification tends to exclude larger-bodied species with lower dispersal capacity due to higher energetic costs and risks associated with using open matrices [32–34], while favoring small, highly mobile species with generalist diets [36]. This directional selection of traits progressively reduces the diversity of functional strategies available, promoting functional homogenization processes that decrease resource-use complementarity and limit functional redundancy, with potential consequences for the stability and resilience of biological assemblages under environmental disturbance [32,37,38].

In cattle-ranching landscapes of the Colombian Amazon, the progressive loss of tree cover generates continuous habitat structure gradients that intensify these mechanisms [14,39,40]. Avian community responses to such gradients are mediated by functional traits such as dispersal capacity, reproductive efficiency, and trophic specialization, resulting in assemblages dominated by generalist species in highly simplified environments [41–43]. Owing to their diversity in body size, diet, and movement strategies, birds are particularly sensitive to variation in landscape structure and serve as effective indicators of habitat transformation effects on biodiversity and ecosystem functioning [44–46]. Moreover, they perform ecological functions closely linked to morphological and trophic traits that determine their role in energy and matter fluxes within natural systems [36,47–50].

Within this conceptual framework, granivorous birds constitute a functional guild particularly suitable for evaluating how the tree cover gradient operates as an environmental filtering mechanism in cattle-ranching landscapes [51]. The presence and abundance of these species are determined by a functional trade-off between the availability of grass and herb seeds in open and semi-open areas and the presence of arboreal components providing refuge, perching sites, and structural connectivity [52]. In highly simplified productive matrices, environmental filtering tends to favor small-bodied, highly mobile granivorous species with flexible diets, such as *Sicalis flaveola* and *Volatinia jacarina* [41], as well as some species of the genus *Sporophila* [53]. In contrast, larger-bodied granivores with lower mobility and greater dependence on dense vegetation, such as the tinamous *Crypturellus cinereus* and *Tinamus guttatus*, tend to be restricted to covers with greater structural complexity [44,54]. This functional reorganization of assemblages illustrates how habitat simplification may reduce available trait space and promote more redundant communities [55], whereas greater tree cover retention may be associated with more functionally diverse assemblages. In this sense, intermediate livestock landscape configurations, such as silvopastoral systems, may represent structural arrangements that balance functional diversity and ecological stability without necessarily maximizing extreme [56,57].

Evaluating these responses requires quantitative approaches integrating multiple dimensions of functional diversity. Multidimensional indices such as functional richness (FRic), functional evenness (FEve), functional divergence (FDiv), functional dispersion (FDis), and Rao’s quadratic entropy (RaoQ) link species functional traits with community assembly processes and potential implications for ecosystem functioning [58–62]. Complementarily, multivariate approaches such as RLQ analysis allow identification of co-structuring patterns among environmental variables, functional traits, and species composition, providing quantitative evidence of trait–environment associations compatible with environmental filtering processes [63].

Accordingly, this study evaluated how the tree cover gradient generated by livestock-driven landscape transformation influences the structure, composition, and functional diversity of granivorous bird communities in cattle-ranching landscapes of the Colombian Amazon. We hypothesized that tree cover acts as an ecological filter structuring the taxonomic and functional diversity of this guild, such that trait combinations associated with structurally complex environments are linked to denser covers, whereas more open covers are associated with species possessing traits compatible with disturbed environments. The study focused on patterns of abundance, taxonomic richness, and trait-based functional structure measured directly in the field along the continuous tree cover gradient. Although functional traits are often discussed in relation to ecological processes and ecosystem services, the present analysis is restricted to empirically observed patterns; any implications regarding ecosystem functions, stability, or resilience are considered theoretical expectations derived from the literature and were not directly evaluated in this research.

## Materials and methods

### Study area

**Fig 1:**
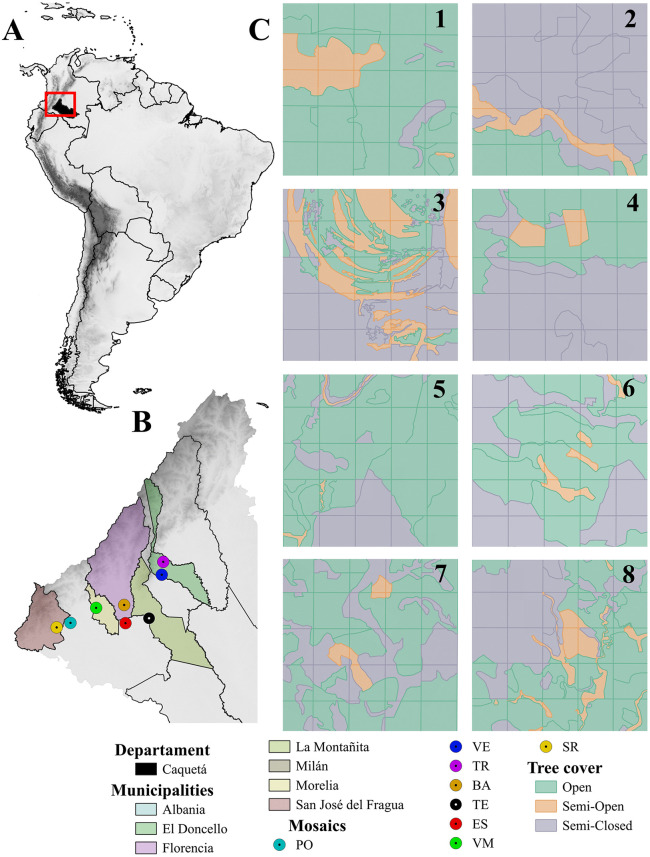
Mosaics of the livestock landscape studied in the Colombian Amazon. Location of the Caquetá department at the national (Colombia) and continental (South America) scale **(A)**; distribution of the eight mosaics across the municipalities of Caquetá **(B)**; classification of the three types of tree cover in the studied mosaics **(C)**; Mosaics: TR: El Triunfo (1); VE: La Vega (2); ES: La Esmeralda (3); TE: El Tesoro (4); VM: Villa Mery (5); PO: El Porvenir (6); SR: Santa Rosa (7); BA: Batalla 13 (8). Base map and imagery: Sentinel-2A (Copernicus Programme, European Space Agency), accessed via the Copernicus Data Space Ecosystem (CDSE). Created by the authors under CC BY 4.0.

The research was conducted in the northwestern region of the Colombian Amazon, across eight livestock landscape mosaics located in seven municipalities within the department of Caquetá: (i) Albania; 1.302766° N – 75.909201° W, 277 m above sea level (a.s.l.), (ii) El Doncello; 1.643959° N – 75.261978° W, 359 m a.s.l., (iii) Florencia; 1.429608° N – 75.528399° W, 248 m a.s.l., (iv) La Montañita; 1.336173° N – 75.352393º W, 269 m a.s.l., (v) Milán; 1.300662° N – 75.519043º W, 222 m a.s.l., (vi) Morelia; 1.409152° N – 75.727271º W, 259 m a.s.l., and (vii) San José del Fragua; 1.270573° N – 76.007303º W, 376 m a.s.l. (Fig 1). The landscapes of the region are characterized by a geomorphology of low hills and piedmont, with floodable valleys and an average slope of 12%. The area has an average temperature of 28.62 °C, relative humidity of 86%, and annual precipitation of 4277 mm, with a unimodal distribution and a peak rainy season from April to October [64].

### Classification of tree cover in livestock landscape mosaics and spatial data preparation

**Fig 2:**
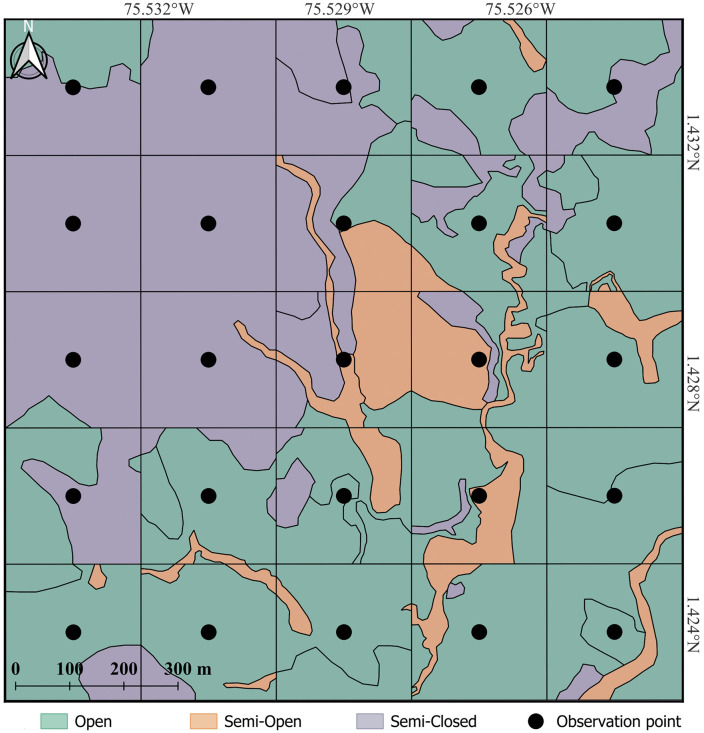
Representation of a mosaic showing the definition of quadrants and the classification of tree cover types, together with bird census observation points in livestock landscapes in the Colombian Amazon. Original figure created by the authors using Sentinel-2A imagery (Copernicus Programme, European Space Agency), accessed through the Copernicus Data Space Ecosystem (CDSE), under CC BY 4.0 license.

Each landscape mosaic corresponded to a square area of 1.56 km² (156 ha), subdivided into 25 symmetrical quadrants of 0.0625 km² (6.25 ha) each (Fig 2). The quadrant constituted the unit of spatial replication for all subsequent environmental and ecological analyses. For each quadrant, a thematic vegetation cover map was generated as the final cartographic product at the unit-of-analysis scale. The base layer was the official Land Cover vector dataset downloaded from the Geoportal of the Territorial Environmental Information System of the Colombian Amazon (SIAT-AC), developed under the CORINE Land Cover methodology adapted for Colombia [65], and its regional adjustment for the Amazon [66], at a nominal cartographic scale of 1:25,000. This product provides polygon-based classification of land-cover types derived from institutional remote-sensing interpretation.

The SIAT-AC vector layer was clipped to the boundaries of each mosaic and subsequently to each quadrant, ensuring that only spatial information strictly corresponding to the sampling units was retained. Because the study required quadrat-level precision (6.25 ha resolution), an additional spatial verification and quality-control procedure was implemented to ensure consistency between institutional cartography and on-site conditions during the 2023 sampling period.

This verification process included: (i) visual inspection of multispectral Sentinel-2A imagery (Copernicus Programme, European Space Agency), accessed via the Copernicus Data Space Ecosystem (CDSE) under a CC BY 4.0 license, using images with cloud cover < 10% and interpreted at an approximate working scale of 1:10,000; and (ii) direct field observations conducted during biological sampling campaigns, aimed at confirming dominant cover classes and detecting transitions among land-cover types.

This step was considered necessary because transitional or heterogeneous covers—such as early secondary regeneration, agroforestry systems, and wetland edges—may exhibit local boundary mismatches or class assignment discrepancies, even when the original product has undergone institutional validation [67]. Such discrepancies can introduce measurement error when environmental predictors are quantified at fine spatial scales.

Following this verification, an edited vector layer (shapefile) was produced by the authors through: (i) geometric adjustment of polygon boundaries when inconsistencies with high-resolution imagery were detected, and/or (ii) punctual reclassification of polygons when field evidence indicated misclassification. All modifications strictly preserved the CORINE/SINCHI classification framework [65,66], avoiding the creation of ad hoc classes. All spatial layers were georeferenced in the MAGNA-SIRGAS 2018 coordinate system (EPSG:9377) and processed using ArcMap version 10.8 [68] and QGIS version 3.40 [69]. From the final edited thematic layer, the area (m^2^) of each polygon within each quadrant was calculated by land-cover class. Tree cover was then quantified a continuous variable at the quadrant scale. Woody vegetation included forest remnants, secondary regeneration patches, tree-dominated agroforestry systems, and arboreal components embedded in pastures, consistent with the structural definition of tree cover under the CORINE framework [65,66]. It is important to clarify that tree cover was not estimated by assigning mean values to discrete land-use categories. Instead, it was directly quantified from the spatially explicit area of polygons representing arboreal vegetation within each sampling quadrat. Consequently, the environmental variable corresponds to an empirical continuous gradient (observed range: 0–60% in this study), derived from polygon-level cartographic quantification and expressed as the proportion of tree cover relative to the total area of each quadrat.

These continuous tree-cover percentages were used directly as environmental predictors in multivariate trait-based analyses (RLQ and fourth-corner tests), without discretization, recoding, or compositional transformation. Because the primary environmental predictor corresponds to a single continuous variable rather than to a closed set of proportions summing to 100%, it is not subject to closed-sum constraints in the multivariate framework.

For descriptive and interpretative purposes only, quadrants were subsequently grouped into three operational structural states based on their observed tree-cover percentages: open (OP), semi-open (SO), and semi-closed (SC) (Table 1). These categories represent ranges along a continuous gradient and do not constitute discrete habitat types. They were used exclusively in analyses of abundance, richness, and taxonomic diversity to facilitate ecologically interpretable contrasts among contrasting structural configurations of livestock landscapes. The full continuous tree-cover values for all quadrants are reported in Supplementary S1 Table, ensuring transparency and enabling independent verification of within-category variability.

**Table 1:** Description of tree cover categories classified within the quadrants of livestock landscape mosaics in the Colombian Amazon.

Tree cover categories	Graphical representation
**Open (OP)** Patches with less than 15% tree vegetation. Pastures sown with *Brachiaria* sp. or *Urochloa* sp. for cattle fodder, and weedy vegetation under 1.5 m in height. Scattered trees and shrubs between 2–5 m tall and trunk diameter (dbh < 10 cm), including species such as *Psidium guajava* (L.), *Bellucia pentamera* (Naudin), *Miconia serrulata* (DC. Naudin), *Cecropia engleriana* (Snethl.), *Zygia longifolia* (Humb. & Bonpl. ex Willd.), and *Inga edulis* (Mart.).	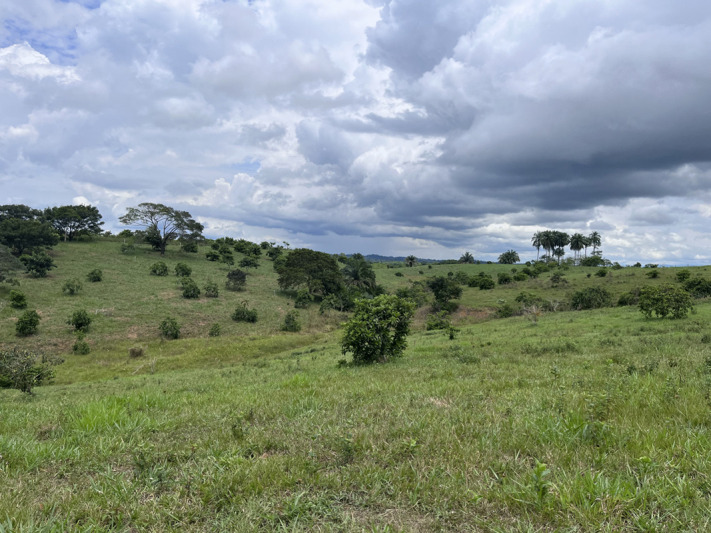
**Semi-Open (SO)** Patches with 15–30% tree vegetation. Agricultural areas and agroforestry systems with cacao (*Theobroma cacao* L.), including tree species such as *Hevea brasiliensis* (Müll. Arg.), *Acacia mangium* (Willd.), *Couma macrocarpa* (Barb. Rodr.), *Citrus limon* (L. Burm. f.), *Cordia alliodora* (Ruiz & Pavón), and *Cedrela odorata* (L.). Includes wetlands and flood-prone zones along alluvial terraces or in the valleys of rolling hills, with aquatic herbs such as *Juncus* (L.).	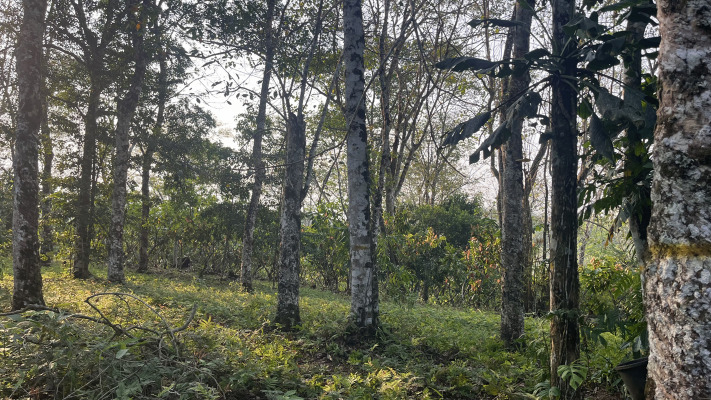
**Semi-Closed (SC)** Patches with 30–60% tree vegetation. Areas with higher vegetation density in early regeneration and succession stages, with pioneer species older than ten years, over 12 m in height, dbh > 15 cm, and an irregular canopy. Species include *Ochroma pyramidale* (Cav. ex Lam. Urb.), *Piptocoma discolor* (Kunth Pruski), *Ormosia coccinea* (Aubl.), *Samanea saman* (Jacq. Merr.), *Aspidosperma excelsum* (Benth.), and *Iriartea deltoidea* (Ruiz & Pav.).	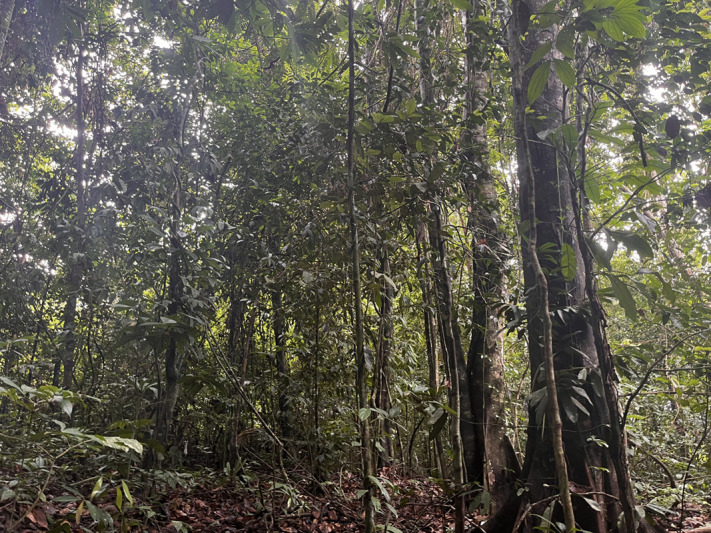

This two-step approach—continuous quantification for inferential modeling and categorical grouping for descriptive comparison—ensures analytical precision while maintaining ecological interpretability, and minimizes bias associated with arbitrary class midpoint assignments or coarse categorical proxies.

### Bird census and functional traits

The granivorous bird assemblage was characterized by combining point counts and mist-net captures conducted between January and November 2023 under standardized protocols of sampling effort, duration, and environmental conditions. Both methods were implemented within controlled temporal windows and at fixed locations within each quadrat to minimize repeated detections and ensure consistency in sampling effort. The quadrat (6.25 ha) constituted the unit of replication for all analyses of abundance, richness, and functional diversity, directly corresponding to the spatial scale at which tree cover was quantified.

Sampling covered nearly a complete annual cycle, incorporating regional climatic variability. Because the species included correspond exclusively to resident granivorous birds, seasonal migration was not expected to significantly influence assemblage composition. Additionally, all landscape mosaics were sampled within the same temporal window, reducing potential seasonal bias and allowing observed patterns to be interpreted primarily along the tree cover gradient.

Observation points were established at the center of each quadrat and spaced 250 m apart, resulting in 25 fixed points per mosaic (Fig 2). Point counts followed standardized protocols widely used in studies of bird communities in tropical and productive landscapes [70,71]. Each count lasted 25 minutes and was restricted to a fixed radius of 50 m around the observer. Only individuals detected visually or acoustically within this radius were recorded. Movement direction, spatial position, and behavioral cues were considered to reduce the probability of recording the same individual more than once, particularly in open habitats with high mobility.

**Fig 3:**
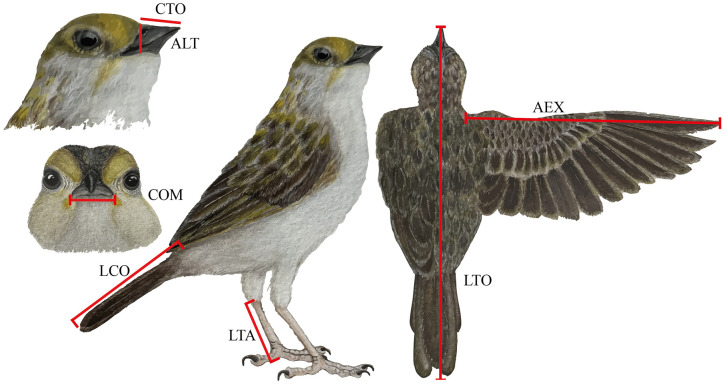
Diagram of the morphological functional traits measured per species. CTO: total culmen length; LTA: tarsus length; LTO: total body length; AEX: extended wing length; LCO: tail length; COM: commissure; ALT: bill height; PES: body weight (g). Illustration of *Ammodramus aurifrons* (Passeriformes: Passerellidae) by Viviana Tello M.

Mist-net sampling consisted of installing four 12 m nets per quadrat, strategically positioned to intercept flight paths between tree-covered areas [43,72]. Nets were operated for five morning hours (06:00–11:00) and checked every 20 minutes. Total mist-netting effort per mosaic was 100 net-hours. Each captured individual was measured for eight morphological traits used to quantify functional diversity (Fig 3): total body length (LTO), tail length (LCO), tarsus length (LTA), extended wing length (AEX), commissure length (COM), bill height (ALT), total culmen length (CTO), and body mass (PES). Measurements were obtained following standardized morphometric procedures.

For species detected but not captured, trait values were obtained from specimens housed in the Ornithological Collection of the Natural History Museum of the Universidad de la Amazonia (UAM), Colombia, and from the global AVONET database [45]. Taxonomic identification followed the nomenclature of the South American Classification Committee [73] and was verified using bird guides for Colombia [74–76]. Species were classified into seven trophic guilds (frugivores, insectivores, nectarivores, granivores, folivores, scavengers, and vertebrate consumers). However, only species assigned to the granivore guild (GRA) were included in this study.

Species-level abundance in each quadrat was estimated through the standardized integration of records obtained from point counts and mist-net captures. To prevent double counting, point-count records were incorporated only when they did not correspond to individuals previously captured in mist nets, based on spatial proximity, timing of detection, and observable characteristics. Temporal coordination between methods further reduced potential overlap. Abundance and richness values represent aggregated counts at the quadrat scale derived from effective sampling effort and confirmed detections, ensuring that each quadrat functions as an independent observational unit.

Although detectability may vary with vegetation structure, the objective of this study was to compare relative differences along a continuous tree cover gradient under a uniform sampling design, rather than to estimate detectability-corrected absolute densities. By standardizing sampling effort, duration, and environmental conditions across quadrats, any residual variation in detectability is assumed to be comparably distributed among units, allowing valid landscape-scale inference in accordance with methodological recommendations on detectability control and standardized avian surveys [70,71,77]. All detections were analyzed at the quadrat scale, ensuring direct correspondence between biological response variables and environmental predictors in subsequent statistical analyses.

### Ethics statement

The specimen collections were conducted under the framework of Resolution 1015 of 2016, in accordance with the General Permit for the Collection of Specimens of Wildlife Species for Non-Commercial Scientific Research, issued to the Universidad de la Amazonia. Approval for practices involving the use of animals was granted by the Institutional Committee on Ethics and Bioethics in Research of the Universidad de la Amazonia (FO-A-APC-01–11), established through Agreement 020 of 2018 by the Superior Council.

### Data analysis

All analyses were conducted using the sampling quadrat (n = 100) as the unit of replication, directly corresponding to the spatial scale at which the continuous percentage of tree cover was quantified. Quadrats were distributed among open (OP, n = 32), semi-open (SO, n = 34), and semi-closed (SC, n = 34) structural categories, which were used exclusively for comparative and descriptive purposes. The continuous percentage of tree cover per quadrat constituted the environmental variable used in the multivariate analyses.

The effect of tree cover on the abundance of individuals and species richness of granivorous birds was evaluated using Generalized Linear Mixed Models with a negative binomial distribution (NB GLMM; nbinom2). Tree cover was included as a fixed effect, and the livestock landscape mosaic in the Colombian Amazon was incorporated as a random effect to account for the hierarchical structure of the design and to avoid pseudoreplication. The adequacy of the distribution was verified through overdispersion diagnostics using DHARMa, confirming the robustness of statistical inference. Post hoc comparisons among OP, SO, and SC categories were performed using Tukey’s method (α = 0.05) in R version 4.2 [78], restricted to ecologically justified contrasts.

Alpha taxonomic diversity was estimated using Hill numbers, which express the effective number of species per quadrat and per tree cover category. Rarefaction–extrapolation curves were generated for the first three orders: q0 (species richness), q1 (Shannon diversity), and q2 (dominance) [79], using the iNEXT.3D package [80], incorporating explicit assessment of sampling completeness. Pooled (γ) diversity was estimated by tree cover type by aggregating all corresponding quadrats (OP = 32; SO = 34; SC = 34), allowing explicit comparison between local (α) and accumulated (γ) diversity patterns without formally attributing mechanisms of spatial turnover.

Functional structure was examined using complementary multivariate analyses. First, hierarchical cluster analysis was conducted using Ward’s algorithm and Gower’s similarity index to group granivorous bird species based on functional trait similarity, and a Principal Component Analysis (PCA) was performed to explore covariation patterns among traits. These procedures were conducted in InfoStat statistical software version 2020 [81]. Differences in morphometric traits among OP, SO, and SC categories, defined at the quadrat scale, were evaluated using General and Mixed Linear Models (GMLM), with traits as response variables and tree cover category as a fixed effect. Model assumptions were verified through graphical inspection of residuals. Post hoc comparisons were conducted using Fisher’s LSD (α = 0.05) only after significant global tests and restricted to ecologically motivated a priori contrasts in R version 4.2 [78], maintaining a balance between Type I error control and statistical power given the limited number of comparisons.

The relationship between environmental variables and functional traits was assessed using RLQ analysis with ade4 package, statistical significance was evaluated using fourth-corner tests (modeltype = 2) [82] in R version 4.2 [78]. The environmental matrix (R) included the proportional area of open (OP), semi-open (SO), and semi-closed (SC) tree cover per quadrat, treated as continuous environmental variables derived from spatial measurements. The L matrix corresponded to granivorous bird abundance per quadrat, and the Q matrix represented species-level functional traits. Matrices R and Q were subjected to Principal Component Analysis (PCA), while matrix L was analyzed using Correspondence Analysis (COA). A co-inertia approach was applied to maximize the covariation between R and Q through L. Statistical significance of environment–trait associations was evaluated using the fourth-corner test with 9,999 permutations, and p-values were adjusted for multiple comparisons using the False Discovery Rate (FDR) procedure [83]. Structural cover categories (OP, SO, SC) were not included as predictors in the RLQ or fourth-corner tests and were used exclusively for descriptive visualization.

Functional diversity at the quadrat scale was quantified using five multidimensional indices [84,85]: FRic (functional richness), FEve (functional evenness), FDiv (functional divergence), FDis (functional dispersion), and RaoQ (quadratic entropy), calculated with the FD package [62] in R version 4.2 [78]. Differences among tree cover categories were evaluated using GMLM, incorporating tree cover as a fixed effect and mosaic as a random effect. Fisher’s LSD (α = 0.05) was applied only following significant global tests. Additionally, a functional diversity analysis based on Area Under the Curve (AUC) was conducted using Hill numbers [86], generating rarefaction–extrapolation curves for q0, q1, and q2 [79,87] with iNEXT.3D [80,88].

Effective sample size varied among analytical blocks due to minimum species richness requirements per quadrat. GLMM analyses of abundance and richness included all 100 quadrats. For functional diversity indices, FRic, FEve, and FDiv were calculated only for quadrats with ≥ 3 species (n = 42; OP = 16, SO = 19, SC = 7), whereas FDis and RaoQ were calculated for quadrats with ≥ 2 species (n = 70; OP = 26, SO = 27, SC = 17). RLQ and fourth-corner analyses included n = 100 quadrats and S = 22 species, using complete community, environmental, and trait matrices. The total number of sampled quadrats and the number included in each index are reported in Supplementary S2 Table.

## Results

A total of 560 individuals of granivorous birds were recorded, representing 22 species, four families, and three orders. The order Passeriformes exhibited the highest species richness and abundance. The species *Volatinia jacarina*, *Patagioenas cayennensis*, and *Ammodramus aurifrons* accounted for approximately 50% of the individuals observed. The complete functional trait matrix is provided in Supplementary S3 and S4 Tables, and the R code along with the data are available in Supplementary S1 Code and S1 Data.

Abundance and species richness were modeled at the quadrat scale (n = 100) using negative binomial generalized linear mixed models (NB GLMM; nbinom2; log link), with tree cover category included as a fixed effect and livestock landscape mosaic incorporated as a random intercept to account for the hierarchical sampling design. Zero-inflated alternatives were explicitly evaluated to assess whether excess zeros influenced model structure; however, no zero counts were observed in the quadrat-level response variables, and standard negative binomial models showed lower AIC values (ΔAIC = 2). Residual diagnostics based on DHARMa simulations indicated no evidence of overdispersion or zero inflation, supporting the adequacy of the selected error distribution for count data under the adopted aggregation scheme.

Because the absence of zero counts is uncommon in guild-level bird data, additional sensitivity analyses were conducted including quadrats with zero detections while maintaining the same model structure and random-effect specification. These alternative models produced consistent effect directions, comparable parameter estimates, and identical inferential conclusions. This confirms that the reported patterns were not driven by data aggregation procedures or by the exclusion of zero observations but rather reflect stable differences across the tree cover gradient under the standardized sampling design.

For abundance, the standard negative binomial NB GLMM was preferred over the zero-inflated alternative (AIC 540.78 vs 542.78). Tree cover had a significant overall effect (Type II Wald χ² = 6.42; df = 2; p = 0.040). Post hoc comparisons indicated that abundance under semi-open cover (SO) was significantly higher than under semi-closed cover (SC) (IRR = 1.69; p = 0.031), whereas other contrasts were not statistically significant. For species richness, the standard negative binomial NB GLMM was also preferred (AIC 380.00 vs 382.00). Tree cover did not show a significant overall effect (Type II Wald χ² = 5.21; df = 2; p = 0.074), indicating that mean species richness per quadrat did not differ significantly among OP, SO, and SC categories.

**Fig 4:**
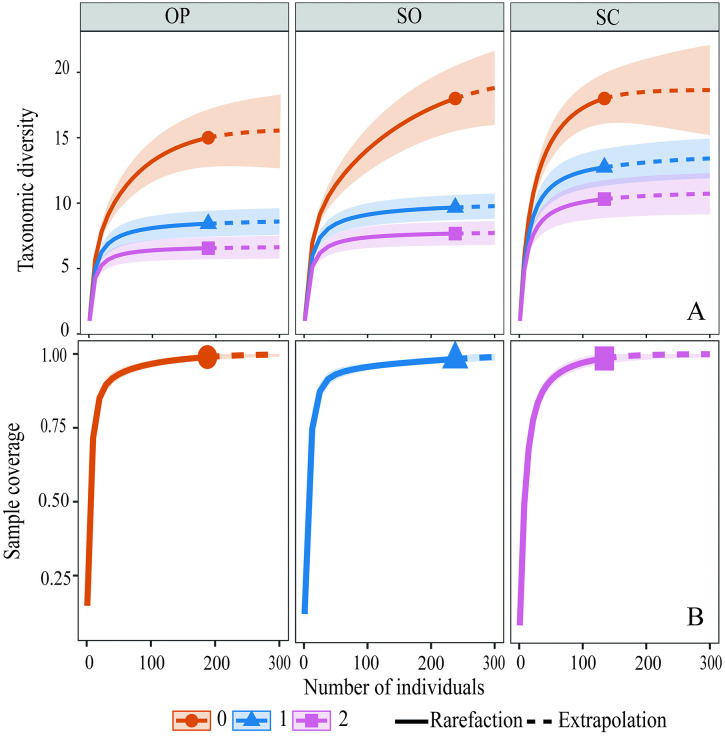
Rarefaction-extrapolation curves of diversity orders q0, q1, and q2 for the granivorous bird assemblage across the tree cover gradient in livestock landscapes of the Colombian Amazon. OP: open; SO: semi-open; SC: semi-closed. A: Sample size-based sampling curve; B: Sample completeness curve.

In contrast to quadrat-level patterns, pooled (γ) diversity estimated by tree cover category using Hill numbers revealed higher accumulated richness under SC. This category exhibited the highest observed richness (q0 = 18 species; n = 134 individuals) and higher values for q1 and q2 (Fig 4A). Sample completeness analysis indicated that the SO category would require approximately three times more individuals (~3×) to reach comparable levels of completeness (Fig 4B). This pattern is consistent with a higher proportion of rare species and/or greater heterogeneity among quadrats within that category, although spatial turnover was not formally partitioned in this study. These results therefore indicate a divergence between local (α) and pooled (γ) diversity patterns across the tree cover gradient.

### Functional classification of species

**Fig 5:**
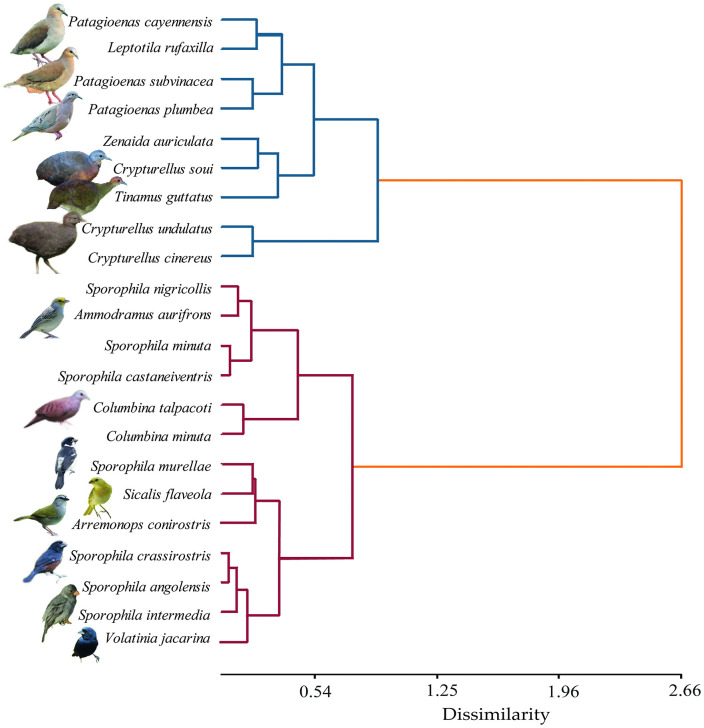
Dendrogram showing the results of the hierarchical cluster analysis using the *ward.D* algorithm. The algorithm’s distance was calculated using the default transformation of the *Gower* similarity index.

The hierarchical cluster analysis (Ward algorithm; Gower similarity index) identified two clearly differentiated functional groups (Fig 5). The first group comprised nine large-bodied granivorous species belonging to Columbiformes and Tinamiformes. The second group consisted of thirteen smaller species, primarily Passeriformes, together with two species of the genus *Columbina*. Functional similarity between groups was below 30%, indicating a clear morphological separation between large and small granivorous birds. The resulting species-by-group matrix is presented in Supplementary S5 Table.

**Fig 6:**
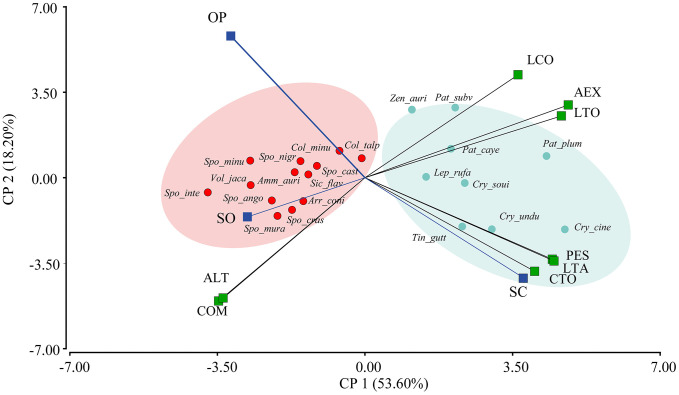
Biplot derived from the principal component analysis of species traits in the granivorous bird assemblage and tree cover types across productive landscapes in the southeastern Colombian Amazon. OP: open; SO: semi-open; SC: semi-closed; CTO: total culmen length; PES: body weight **(g)**; LTA: tarsus length; LTO: total body length; AEX: extended wing length; LCO: tail length; COM: commissure; ALT: bill height.

Principal component analysis (PCA) explained 71.8% of the total variance across the first two axes. Along PC1, larger-bodied and heavier species were associated with higher percentages of tree cover (semi-closed sites), whereas smaller species with broader and taller bills were associated with open and semi-open covers (Fig 6). The second axis (PC2) reflected variation in locomotor and trophic structures without strict separation among cover types.

At the quadrant scale, several morphological traits showed significant differences among tree cover categories: total culmen length (CTO: F = 6.69; p = 0.0015), extended wing length (AEX: F = 3.56; p = 0.0297), total body length (LTO: F = 3.82; p = 0.0232), and body weight (PES: F = 6.52; p = 0.0017), with higher mean values observed under semi-closed cover. Other traits showed no statistical evidence of differences, indicating that morphological differentiation across the tree cover gradient was selective rather than uniform across all trait dimensions at the quadrat scale.

### Trait–Cover association (RLQ and Fourth-Corner tests)

**Fig 7:**
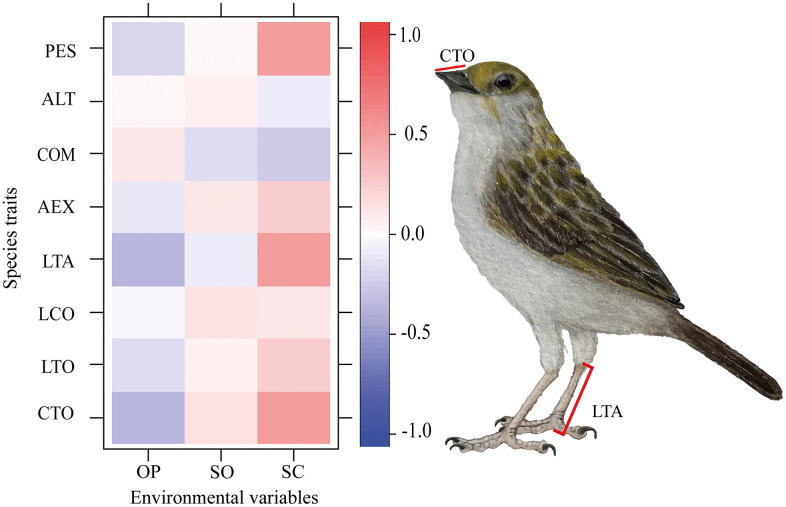
RLQ analysis between the environmental matrices of the percentage of the three types of tree cover and the functional traits of granivorous bird species in cattle ranching landscapes of the Colombian Amazon. ALT: bill height; COM: commissure; AEX: extended wing; LTA: tarsus length; LCO: tail length; LTO: total body length; CTO: total culmen length; PES: body weight (g); OP: open; SO: semi-open; SC: semi-closed. Red squares indicate positive associations, while blue squares indicate negative associations. Lighter colors indicate weaker associations, whereas darker colors represent stronger associations. The illustration of *Ammodramus aurifrons* highlights the traits with the strongest associations.

The RLQ analysis assessed the co-structure among the environmental matrix (R), composed of the proportions of tree cover in the OP, SO, and SC categories, the species abundance matrix (L), and the species-level functional trait matrix (Q), weighted by abundance. The first two canonical axes accounted for 100% of the total co-structural inertia, with RLQ1 explaining 93.42% and RLQ2 6.58% of the total covariance (Fig 7). These values represent the relative distribution of the shared variance among R, L, and Q. The first RLQ axis captured the dominant pattern of association between tree cover structure and species functional traits. The global Monte Carlo test was significant (p = 0.0245; 9,999 permutations), indicating that the observed co-structure exceeded that expected under the null model.

These patterns were statistically confirmed using the fourth-corner test. Significant correlations were identified (p < 0.05, FDR-adjusted) between the percentage of tree cover and functional traits. In particular, a significant negative association was detected between open cover and the functional traits total culmen length (CTO; r = –0.1840; p = 0.0032), tarsus length (LTA; r = –0.1703; p = 0.0105), total body length (LTO; r = –0.1335; p = 0.0340), and body mass (PES; r = –0.1619; p = 0.0097). In contrast, commissure width was positively correlated with open cover (COM; r = 0.1274; p = 0.0251).

Accordingly, smaller-bodied and lighter granivorous birds, characterized by shorter and wider bills and shorter tarsi, were associated with areas exhibiting lower percentages of tree cover (OP). Conversely, larger-bodied granivorous birds (LTO; r = 0.1250; p = 0.0496) and heavier species (PES; r = 0.1718; p = 0.0063), with longer tarsi (LTA; r = 0.1929; p = 0.0032) and longer (CTO; r = –0.1840; p = 0.0032) and more slender bills (COM; r = –0.1238; p = 0.0278), were associated with areas exhibiting higher percentages of tree cover. These results indicate statistically significant trait–environment covariation along the continuous tree cover gradient. The R code along with the data are available in the Supplementary Material S2 Code and S2 Data.

### Functional diversity of the granivorous bird assemblage

At the quadrant scale, only FDis (F = 7.12; p = 0.0013; df = 2) and RaoQ (F = 7.41; p = 0.0001; df = 2) showed significant differences among cover categories. FDis peaked under semi-open cover, whereas RaoQ was highest under semi-closed cover. Fric and FEve did not show significant differences. The R code along with the data are available in the Supplementary Material S3 Code and S3 Data

**Fig 8:**
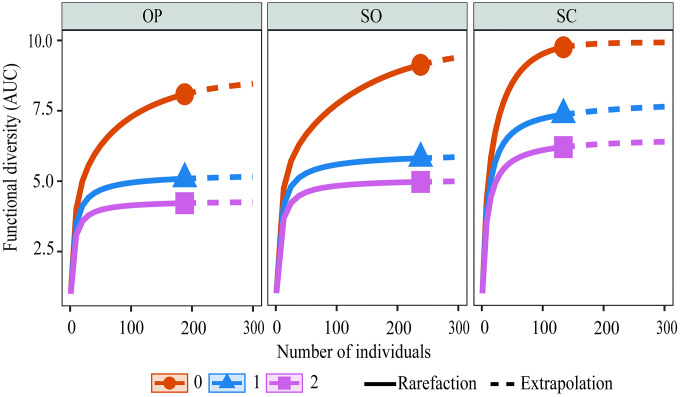
Rarefaction–extrapolation curves of functional diversity of order q0, q1, and q2 for the granivorous bird assemblage across the tree cover gradient in cattle ranching landscapes of the Colombian Amazon. OP: open; SO: semi-open; SC: semi-closed.

In the AUC-based functional diversity analysis, rarefaction–extrapolation curves indicated higher q0, q1, and q2 values under semi-closed cover at reduced sample sizes (Fig 8). When extrapolated to larger sampling sizes, functional diversity indices increase particularly in open and semi-closed covers. These patterns reflect scaling properties of accumulated functional diversity rather than changes in per-quadrat functional structure, indicating statistical association between structural complexity and scaling patterns of functional diversity.

The results indicate that different metrics capture complementary dimensions of community assembly: local abundance reaches its maximum under intermediate tree cover, cumulative species richness is higher in semi-closed cover, and functional dispersion peaks in structurally intermediate states. These findings indicate that the observed patterns are not monotonic but instead reflect dimension-specific differentiation across the tree cover gradient.

## Discussion

The results demonstrate a statistically significant effect of tree cover type on the mean abundance of the granivorous bird assemblage at the quadrat scale, whereas species richness exhibited only a marginal overall response. Consequently, variation along the tree cover gradient is primarily expressed through changes in the number of individuals rather than consistent modifications in the number of species per sampling unit. Although semi-open covers showed higher mean values for both abundance and richness, only abundance displayed statistically supported differences among cover types. Therefore, the observed trend in richness should be interpreted descriptively and not as conclusive evidence of a consistent effect of the structural gradient on local taxonomic diversity. In this context, the results suggest that structural differences in the landscape are mainly associated with variation in assemblage magnitude, expressed as abundance, rather than systematic shifts in taxonomic composition at the local scale.

The sampling design, implemented simultaneously across mosaics and extended over nearly one year, allowed integration of the annual climatic variability characteristic of the Colombian Amazon and reduced potential seasonal biases. Moreover, by focusing exclusively on resident species within the granivorous guild, it is unlikely that the observed patterns were influenced by migratory movements. These methodological characteristics strengthen the interpretation of the findings as consistent associations within an observational framework. Comparable patterns have been documented in tropical agricultural landscapes, where intermediate structural configurations are associated with higher abundances without necessarily implying increases in local richness [89–94]. In the present study, however, these parallels are presented as contextual comparisons rather than direct evidence of specific ecological mechanisms.

Species such as *Volatinia jacarina*, *Patagioenas cayennensis*, and *Ammodramus aurifrons* were the most abundant during sampling. These species were primarily recorded in open and semi-open covers dominated by pastures, vegetation edges, and early successional stages. The literature indicates that their functional trait combinations are associated with efficient resource exploitation in habitats with low tree cover: *V. jacarina* in anthropogenic grasslands [95,96]; *P. cayennensis* in fragmented landscapes [35,97]; and *A. aurifrons* in livestock pastures dominated by forage grasses [53,98]. These patterns are consistent with previous studies [41,99,100] describing associations between reduced vertical complexity and higher population densities in these species. However, such responses should be interpreted at the assemblage level—as expressions of disturbance tolerance, niche breadth, and ecological plasticity—rather than as evidence of strict or exclusive specialization to open environments [31,45,46,101–103].

Within this framework, the patterns observed along the tree cover gradient are consistent with the multivariate structure of avian dietary space described by Barnagaud et al. [104], in which granivory is frequent in structurally simplified and human-modified environments. From this perspective, the higher abundance of granivorous birds in semi-open covers may reflect environmental filtering along structural gradients, consistent with theoretical expectations. Accordingly, the RLQ analysis revealed statistically significant covariation between tree cover percentage and morphological traits, particularly body size and bill morphology, indicating functional structuring associated with the environmental gradient [32–34]. Nevertheless, these findings describe covariation patterns and do not demonstrate causal mechanisms or deterministic specialization by cover type [105,106].

The tree cover gradient was also associated with differential occurrence of certain granivorous species. Some species were detected exclusively in a single cover type, such as *Zenaida auriculata* in open areas and *Sporophila nigricollis*, *S. intermedia*, and *S. minuta* in semi-open covers. In contrast, larger-bodied species such as *Crypturellus cinereus* and *Tinamus guttatus* were detected exclusively in semi-closed covers. These patterns may reflect differences in structural habitat requirements [33–35]; however, they should be interpreted cautiously, as detectability may vary with structural complexity and population density [25,231, 42,97]. In the case of *T. guttatus*, classified as “Near Threatened” [107], its association with more complex covers suggests the potential importance of these remnants for local persistence [108,109], although this study did not explicitly evaluate demographic parameters or population persistence probabilities.

Hierarchical cluster analysis based on morphological traits identified two main functional groups within the granivorous guild: one composed of larger-bodied species, including *P. plumbea*, *P. cayennensis*, *C. soui*, and *T. guttatus*, and another comprising smaller-bodied species, including representatives of the genera *Sporophila* and *Columbina*. This differentiation was consistent with patterns revealed by PCA and RLQ analyses, which showed statistical associations between traits such as culmen length (CTO), tarsus length (LTA), and body mass (PES) and semi-closed covers. These associations indicate that certain trait combinations are more represented in structurally complex environments [110–115], without implying direct evidence of adaptive advantages or differential performance [50,116,117]. In contrast, open covers were associated with smaller-bodied species and relatively wider, deeper bills, linked to diets dominated by Poaceae and Cyperaceae seeds, including forage grasses such as *Brachiaria* sp., widely available in livestock systems [118–120]. Given that multiple studies have documented heterogeneous and context-dependent responses to disturbance gradients [121–124], these findings are presented as structural assemblage trends.

Overall, variation in tree cover within Amazonian livestock mosaics was statistically associated with changes in abundance patterns and specific dimensions of the functional structure of the granivorous guild. These associations are consistent with theoretical expectations of environmental filtering [31–33,37,41,51]; however, the study did not directly evaluate underlying mechanisms. Reduced tree cover was linked to decreases in abundance-weighted functional dispersion indices (FDis and RaoQ), whereas other indices such as FRic and FEve did not show significant differences. Collectively, the results suggest partial reorganization of functional space, compatible with patterns of functional simplification or homogenization documented in transformed systems [49,125–130], but they do not indicate a uniform contraction of total functional volume.

### Functional diversity of the granivorous assemblage

From the conceptual framework of environmental filtering, the response of functional diversity to the tree cover gradient may be interpreted as consistent with the potential action of hierarchical filters associated with livestock landscape configuration [33,131–137]. In the Colombian Amazon, these landscapes are characterized by open matrices dominated by pastures, with scattered remnant vegetation patches and reduced functional connectivity [7,43,138,139], conditions associated with functional space restructuring and shifts in trait distribution [140].

The results indicate that different functional diversity indices capture complementary dimensions of the assemblage and respond non-uniformly to the structural gradient. FDis and RaoQ showed statistically significant differences among cover types, indicating changes in trait dispersion and dominance structure weighted by abundance. In contrast, FRic and FEve did not differ significantly, suggesting stability in total functional volume and trait space evenness. Semi-open covers exhibited relatively higher FDis values, reflecting greater abundance-weighted functional dispersion, whereas semi-closed covers showed descriptively higher FRic and FEve values, although without conclusive statistical support. These patterns are consistent with studies documenting functional reorganization under landscape transformation without necessarily implying expansion or contraction of total functional volume [34,44,104,141].

Open-cover mosaics displayed the lowest functional diversity values, consistent with the structural simplification characteristic of Amazonian livestock matrices [43,64,142–144]. Several studies have linked the dominance of small, opportunistic species with increased functional redundancy in simplified environments [9,29,100,145]. Previous research has also suggested that loss of ecosystem processes, including seed dispersal and vegetation regeneration, may be associated with functional homogenization [123,146–149]; however, such processes were not evaluated in this study and should be considered theoretical implications derived from the literature.

In summary, functional diversity within the granivorous guild was associated with the tree cover gradient through reorganization of trait dominance and abundance structure, consistent with structural trends observed in transformed landscapes [150–157]. These patterns should be interpreted as observational associations rather than deterministic rules. From an applied perspective, the findings suggest that landscape configurations with greater structural heterogeneity may favor higher abundance-weighted functional dispersion within the granivorous guild [151,153,154]. Nevertheless, the study did not directly assess livestock productivity, ecosystem service provision, or long-term sustainability; therefore, such implications should be regarded as hypotheses for future research derived from the present findings.

## Conclusions

According to the study findings, the continuous tree cover gradient was significantly associated with the abundance of the granivorous bird assemblage at the quadrat scale, whereas taxonomic richness exhibited only marginal responses. This pattern indicates that structural variation in the landscape is expressed primarily through changes in the number of individual birds rather than through consistent modifications in the number of species per sampling unit. From a functional perspective, the reorganization of trait space was partial and non-uniform. Differences observed in certain functional diversity indices among cover types suggest changes in the dispersion and relative dominance of abundance-weighted functional traits, whereas other dimensions of the functional volume remained statistically stable. Consistently, significant multivariate covariation was detected between proportions of tree cover and morphological traits associated with body size and bill and tarsus morphology in granivorous birds. Overall, these results are consistent with theoretical expectations of environmental filtering along structural gradients. However, given the observational nature of the study, these findings should be interpreted as empirical associations rather than causal evidence or direct demonstrations of effects on ecosystem functions.

## Supporting information

S1 Table Total percentage of tree cover for each of the three classified categories within the 100 quadrants established across the eight livestock landscapes mosaics in the Colombian Amazon. (DOCX)

S2 Table Effective sample size per functional diversity index. (DOCX)

S3 Table List and matrix of morphological functional traits of the 22 granivorous bird species recorded across the three types of tree cover. (DOCX)

S4 Table Matrix of morphological traits for each granivorous bird species recorded in the study. (DOCX)

S5 Table Matrix of granivorous bird species assigned to two groups (small and large) based on the dendrogram generated by hierarchical cluster analysis using the ward.D algorithm. (DOCX)

S1 Code R code of the GLMM with mixed-effects negative binomial regression model to evaluate the effect of tree cover types on the richness and abundance of granivorous birds. (R)

S2 Code R Code for RLQ and Fourth Corner analysis between environmental matrices of tree cover types and functional traits of granivorous birds. (R)

S3 Code R Code of the General and Mixed Linear Models (GMLM) of functional diversity indices of granivorous birds across three types of tree cover. (R)

S1 Data Data matrix used in the R code for GLMM analysis with mixed-effects negative binomial regression model. (TXT)

S2 Data Environmental, species, and trait matrices for the R code for RLQ and Fourth Corner analysis. (XLSX)

S3 Data Data matrix used in the R code for GMLM of functional diversity indices. (CSV)
