# Supplementary material for: Livestock landscapes as ecological filters: Effects of the tree cover gradient on the taxonomic and functional diversity of granivorous birds in the Colombian Amazon
Source: PLoS One. 2026 Mar 20;21(3):e0345283. doi: 10.1371/journal.pone.0345283 (PMC13004383; doi:10.1371/journal.pone.0345283)
Supplement: S1 Table — (DOCX) [file pone.0345283.s001.docx]

**S1 Table.** Total percentage of tree Cover for each of the three classified categories within the 100 quadrants established across theeight livestock landscapes mosaics in the Colombian Amazon.

| **Mosaics** | **Quadrant code** | **% Tree cover** | | | **% Total tree cover per quadrant** | **Tree cover type by quadrant** | **Latitude** | **Longitude** |
| --- | --- | --- | --- | --- | --- | --- | --- | --- |
|  |  | **OP** | **SO** | **SC** |  |  |  |  |
| BA | BA01 | 8.20% | 0.56% | 9.81% | 18.58% | SO | 1.42383 | -75.53331 |
|  | BA03 | 9.37% | 2.21% | 0.00% | 11.58% | OP | 1.42384 | -75.52882 |
|  | BA04 | 8.68% | 4.21% | 0.69% | 13.58% | OP | 1.42384 | -75.52657 |
|  | BA05 | 8.28% | 4.50% | 2.63% | 15.40% | OP | 1.42384 | -75.52433 |
|  | BA07 | 6.58% | 10.56% | 2.43% | 19.56% | SO | 1.4261 | -75.52657 |
|  | BA08 | 7.44% | 5.01% | 6.77% | 19.22% | SO | 1.4261 | -75.52882 |
|  | BA09 | 8.11% | 1.36% | 9.01% | 18.48% | SO | 1.4261 | -75.53107 |
|  | BA10 | 5.03% | 0.00% | 29.81% | 34.84% | SC | 1.42609 | -75.53331 |
|  | BA12 | 0.00% | 0.78% | 58.66% | 59.44% | SC | 1.42836 | -75.53107 |
|  | BA13 | 1.05% | 16.94% | 24.69% | 42.67% | SC | 1.42836 | -75.52882 |
|  | BA14 | 4.37% | 15.52% | 7.21% | 27.09% | SO | 1.42836 | -75.52658 |
|  | BA15 | 8.43% | 5.51% | 0.00% | 13.94% | OP | 1.42836 | -75.52433 |
|  | BA16 | 9.34% | 0.43% | 3.24% | 13.00% | OP | 1.43062 | -75.52433 |
|  | BA17 | 6.32% | 4.50% | 14.38% | 25.19% | SO | 1.43062 | -75.53107 |
|  | BA18 | 2.50% | 9.56% | 28.59% | 40.65% | SC | 1.43062 | -75.52883 |
|  | BA19 | 0.00% | 0.00% | 60.00% | 60.00% | SC | 1.43062 | -75.52658 |
| ES | ES02 | 0.00% | 0.00% | 60.00% | 60.00% | SC | 1.29707 | -75.52305 |
|  | ES03 | 0.00% | 3.39% | 54.19% | 57.58% | SC | 1.29612 | -75.52101 |
|  | ES06 | 0.00% | 2.74% | 55.30% | 58.04% | SC | 1.29628 | -75.51599 |
|  | ES07 | 0.02% | 10.92% | 41.16% | 52.10% | SC | 1.29723 | -75.51803 |
|  | ES12 | 2.83% | 21.18% | 6.68% | 30.70% | SC | 1.30118 | -75.52117 |
|  | ES14 | 3.19% | 9.02% | 25.40% | 37.61% | SC | 1.29928 | -75.51709 |
| PO | PO03 | 8.09% | 0.00% | 11.44% | 19.54% | SO | 1.29826 | -75.90795 |
|  | PO04 | 4.06% | 0.00% | 35.64% | 39.70% | SC | 1.29877 | -75.90577 |
|  | PO05 | 4.56% | 0.00% | 32.67% | 37.22% | SC | 1.29927 | -75.90358 |
|  | PO06 | 0.00% | 0.00% | 60.00% | 60.00% | SC | 1.30147 | -75.90409 |
|  | PO07 | 5.68% | 2.97% | 20.83% | 29.48% | SO | 1.30097 | -75.90627 |
|  | PO09 | 7.08% | 0.29% | 17.01% | 24.38% | SO | 1.29996 | -75.91065 |
|  | PO11 | 7.04% | 0.00% | 17.77% | 24.81% | SO | 1.30165 | -75.91335 |
|  | PO12 | 8.19% | 2.15% | 7.16% | 17.50% | SO | 1.30216 | -75.91116 |
|  | PO13 | 8.66% | 4.68% | 0.00% | 13.35% | OP | 1.30266 | -75.90897 |
|  | PO14 | 8.48% | 3.64% | 2.91% | 15.02% | OP | 1.30317 | -75.90678 |
|  | PO15 | 6.97% | 0.00% | 18.21% | 25.17% | SO | 1.30367 | -75.9046 |
|  | PO16 | 5.16% | 0.00% | 29.06% | 34.22% | SC | 1.30588 | -75.9051 |
|  | PO17 | 9.01% | 3.48% | 0.00% | 12.49% | OP | 1.30537 | -75.90729 |
|  | PO18 | 9.54% | 0.62% | 1.67% | 11.84% | OP | 1.30486 | -75.90948 |
|  | PO19 | 9.09% | 0.00% | 5.48% | 14.56% | OP | 1.30436 | -75.91167 |
|  | PO22 | 4.14% | 0.00% | 35.14% | 39.28% | SC | 1.30656 | -75.91218 |
|  | PO23 | 7.32% | 0.00% | 16.08% | 23.40% | SO | 1.30707 | -75.90999 |
|  | PO24 | 6.14% | 1.88% | 19.95% | 27.97% | SO | 1.30757 | -75.9078 |
|  | PO25 | 8.29% | 2.52% | 5.95% | 16.76% | SO | 1.30808 | -75.90561 |
| SR | SR02 | 7.80% | 0.00% | 13.21% | 21.01% | SO | 1.27337 | -76.01135 |
|  | SR05 | 4.58% | 0.00% | 32.50% | 37.08% | SC | 1.27653 | -76.00539 |
|  | SR06 | 8.18% | 0.00% | 10.91% | 19.09% | SO | 1.27453 | -76.00434 |
|  | SR15 | 3.43% | 0.00% | 39.45% | 42.87% | SC | 1.27253 | -76.0033 |
|  | SR18 | 2.50% | 7.58% | 32.01% | 42.09% | SC | 1.26843 | -76.00623 |
|  | SR21 | 1.89% | 0.00% | 48.64% | 50.53% | SC | 1.26432 | -76.00915 |
| TE | TE01 | 0.00% | 0.00% | 60.00% | 60.00% | SC | 1.32713 | -75.35627 |
|  | TE02 | 0.00% | 0.00% | 60.00% | 60.00% | SC | 1.32904 | -75.35746 |
|  | TE04 | 0.00% | 0.00% | 60.00% | 60.00% | SC | 1.33287 | -75.35985 |
|  | TE05 | 0.00% | 0.00% | 60.00% | 60.00% | SC | 1.33478 | -75.36105 |
|  | TE06 | 0.00% | 0.00% | 60.00% | 60.00% | SC | 1.33599 | -75.35915 |
|  | TE08 | 0.00% | 0.00% | 60.00% | 60.00% | SC | 1.33216 | -75.35676 |
|  | TE10 | 0.00% | 0.00% | 60.00% | 60.00% | SC | 1.32833 | -75.35436 |
|  | TE12 | 1.23% | 0.00% | 52.61% | 53.84% | SC | 1.33145 | -75.35366 |
|  | TE14 | 3.70% | 0.00% | 37.78% | 41.48% | SC | 1.33096 | -75.35866 |
|  | TE15 | 3.70% | 0.00% | 37.78% | 41.48% | SC | 1.33719 | -75.35725 |
|  | TE17 | 3.86% | 18.69% | 4.79% | 27.34% | SO | 1.33648 | -75.35415 |
|  | TE21 | 10.00% | 0.00% | 0.00% | 10.00% | OP | 1.33195 | -75.34866 |
|  | TE23 | 7.51% | 5.81% | 4.96% | 18.29% | SO | 1.33577 | -75.35105 |
|  | TE24 | 8.49% | 0.27% | 8.58% | 17.34% | SO | 1.33769 | -75.35225 |
| TR | TR01 | 10.00% | 0.00% | 0.00% | 10.00% | OP | 1.731 | -75.25589 |
|  | TR02 | 10.00% | 0.00% | 0.00% | 10.00% | OP | 1.73064 | -75.25367 |
|  | TR03 | 10.00% | 0.00% | 0.00% | 10.00% | OP | 1.73028 | -75.25145 |
|  | TR04 | 8.59% | 0.00% | 8.48% | 17.06% | SO | 1.72992 | -75.24923 |
|  | TR06 | 8.75% | 0.00% | 7.49% | 16.24% | SO | 1.73179 | -75.24666 |
|  | TR07 | 6.62% | 0.00% | 20.26% | 26.88% | SO | 1.73215 | -75.24887 |
|  | TR08 | 10.00% | 0.00% | 0.00% | 10.00% | OP | 1.73251 | -75.25109 |
|  | TR09 | 10.00% | 0.00% | 0.00% | 10.00% | OP | 1.73287 | -75.25331 |
|  | TR10 | 10.00% | 0.00% | 0.00% | 10.00% | OP | 1.73323 | -75.25553 |
|  | TR11 | 4.51% | 19.22% | 0.00% | 23.73% | SO | 1.73546 | -75.25517 |
|  | TR13 | 9.27% | 2.56% | 0.00% | 11.83% | OP | 1.73474 | -75.25073 |
|  | TR14 | 9.98% | 0.00% | 0.14% | 10.12% | OP | 1.73438 | -75.24852 |
|  | TR15 | 9.13% | 0.00% | 5.21% | 14.34% | OP | 1.73402 | -75.2463 |
|  | TR16 | 9.16% | 0.00% | 5.06% | 14.22% | OP | 1.73625 | -75.24594 |
|  | TR17 | 9.31% | 0.00% | 4.17% | 13.47% | OP | 1.73661 | -75.24816 |
|  | TR18 | 6.70% | 11.54% | 0.00% | 18.24% | SO | 1.73697 | -75.25038 |
|  | TR19 | 2.63% | 25.80% | 0.00% | 28.43% | SO | 1.73733 | -75.25259 |
|  | TR20 | 3.25% | 23.64% | 0.00% | 26.88% | SO | 1.73769 | -75.25481 |
|  | TR21 | 10.00% | 0.00% | 0.00% | 10.00% | OP | 1.73993 | -75.25445 |
|  | TR22 | 10.00% | 0.00% | 0.00% | 10.00% | OP | 1.73957 | -75.25224 |
|  | TR24 | 10.00% | 0.00% | 0.00% | 10.00% | OP | 1.73884 | -75.2478 |
|  | TR25 | 10.00% | 0.00% | 0.00% | 10.00% | OP | 1.73848 | -75.24558 |
| VE | VE01 | 10.00% | 0.00% | 0.00% | 10.00% | OP | 1.64117 | -75.26804 |
|  | VE02 | 10.00% | 0.00% | 0.00% | 10.00% | OP | 1.64025 | -75.26599 |
|  | VE03 | 7.06% | 0.27% | 17.14% | 24.48% | SO | 1.63932 | -75.26394 |
|  | VE14 | 0.00% | 0.00% | 60.00% | 60.00% | SC | 1.64251 | -75.26004 |
|  | VE15 | 0.00% | 0.00% | 60.00% | 60.00% | SC | 1.64158 | -75.258 |
|  | VE19 | 0.00% | 0.00% | 60.00% | 60.00% | SC | 1.64643 | -75.26322 |
|  | VE23 | 0.00% | 0.00% | 60.00% | 60.00% | SC | 1.64756 | -75.26025 |
| VM | VM01 | 8.01% | 0.00% | 11.92% | 19.93% | SO | 1.41317 | -75.73157 |
|  | VM02 | 8.76% | 0.00% | 7.45% | 16.21% | SO | 1.41091 | -75.73157 |
|  | VM03 | 7.03% | 0.00% | 17.84% | 24.87% | SO | 1.40865 | -75.73157 |
|  | VM04 | 9.80% | 0.00% | 1.20% | 11.00% | OP | 1.40639 | -75.73156 |
|  | VM05 | 7.23% | 3.67% | 10.30% | 21.20% | SO | 1.40413 | -75.73156 |
|  | VM07 | 7.31% | 2.26% | 12.27% | 21.84% | SO | 1.40639 | -75.72932 |
|  | VM08 | 6.66% | 0.00% | 20.02% | 26.68% | SO | 1.40865 | -75.72932 |
|  | VM17 | 8.88% | 0.00% | 6.72% | 15.60% | OP | 1.4064 | -75.72483 |
|  | VM18 | 10.00% | 0.00% | 0.00% | 10.00% | OP | 1.40866 | -75.72483 |
|  | VM19 | 10.00% | 0.00% | 0.00% | 10.00% | OP | 1.41092 | -75.72483 |

OP: open; SO: semi-open; SC: semi-closed.
