# Supplementary material for: Livestock landscapes as ecological filters: Effects of the tree cover gradient on the taxonomic and functional diversity of granivorous birds in the Colombian Amazon
Source: PLoS One. 2026 Mar 20;21(3):e0345283. doi: 10.1371/journal.pone.0345283 (PMC13004383; doi:10.1371/journal.pone.0345283)
Supplement: S2 Table — (DOCX) [file pone.0345283.s002.docx]

**S2 Table. Effective sample size per functional diversity index.** Expresses the total number of sampled quadrants and the number of quadrants for each validly calculated functional diversity index and the number of quadrants excluded due to methodological restrictions (mainly minimum species richness).

| **FDI** | **Total number quadrants** | **Quadrants species detection** | **Minimum species requirement** | **Quadrants calculable index** | **Tree cover** | | | **Excluded quadrants** | **Exclusion criteria** |
| --- | --- | --- | --- | --- | --- | --- | --- | --- | --- |
|  |  |  |  |  | **OP** | **SO** | **SC** |  |  |
| FRic | 200 | 100 | ≥ 3 species | 42 | 16 | 19 | 7 | 58 | Insufficient richness |
| FEve | 200 | 100 | ≥ 3 species | 42 | 16 | 19 | 7 | 58 | Insufficient richness |
| FDiv | 200 | 100 | ≥ 3 species | 42 | 16 | 19 | 7 | 58 | Insufficient richness |
| FDis | 200 | 100 | ≥ 2 species | 70 | 26 | 27 | 17 | 30 | Insufficient richness |
| RaoQ | 200 | 100 | ≥ 2 species | 70 | 26 | 27 | 17 | 30 | Insufficient richness |
| CWM (individual traits) | 200 | 100 | ≥ 1 species | 100 | 32 | 34 | 34 | 0 | Not applicable |

FDI: functional diversity index; FRic: functional richness; FEve: functional evenness; FDiv: functional divergence; FDis: functional dispersión; RaoQ: quadratic entropy; OP: open; SO: semi-open; SC: semi-closed.
