# Supplementary material for: Livestock landscapes as ecological filters: Effects of the tree cover gradient on the taxonomic and functional diversity of granivorous birds in the Colombian Amazon
Source: PLoS One. 2026 Mar 20;21(3):e0345283. doi: 10.1371/journal.pone.0345283 (PMC13004383; doi:10.1371/journal.pone.0345283)
Supplement: S3 Table — (DOCX) [file pone.0345283.s003.docx]

**S3 Table.** List and matrix of morphological functional traits of the 22 granivorous bird species recorded across the three types of tree cover in livestock landscapes of the Colombian Amazon.

| **Order** | **Family** | **Scientific name** | **Mosaics** | **Quadrant** | **TC** | **AB** | **Morphological functional traits** | | | | | | | |
| --- | --- | --- | --- | --- | --- | --- | --- | --- | --- | --- | --- | --- | --- | --- |
|  |  |  |  |  |  |  | **CTO** | **LTO** | **LCO** | **LTA** | **AEX** | **COM** | **ALT** | **PES** |
| Columbiformes | Columbidae | *Columbina minuta* | BA | BA07 | SO | 1 | 15.25 | 147.00 | 57.60 | 12.50 | 119.00 | 10.91 | 3.71 | 31.10 |
|  |  |  | BA | BA16 | OP | 4 | 16.03 | 148.08 | 55.22 | 11.75 | 122.63 | 5.99 | 3.43 | 35.47 |
|  |  |  | TE | TE05 | SC | 4 | 16.03 | 148.08 | 55.22 | 11.75 | 122.63 | 5.99 | 3.43 | 35.47 |
|  |  |  | TE | TE15 | SC | 3 | 16.03 | 148.08 | 55.22 | 11.75 | 122.63 | 5.99 | 3.43 | 35.47 |
|  |  |  | TR | TR03 | OP | 1 | 16.03 | 148.08 | 55.22 | 11.75 | 122.63 | 5.99 | 3.43 | 35.47 |
|  |  |  | TR | TR15 | OP | 1 | 20.17 | 141.03 | 54.39 | 10.17 | 114.83 | 4.05 | 3.70 | 34.20 |
|  |  |  | VM | VM07 | SO | 1 | 12.66 | 156.20 | 53.67 | 12.59 | 134.06 | 3.02 | 2.87 | 41.10 |
|  |  | *Columbina talpacoti* | BA | BA01 | SO | 1 | 18.01 | 158.01 | 65.02 | 12.79 | 133.89 | 4.54 | 4.43 | 43.18 |
|  |  |  | BA | BA12 | SC | 4 | 18.01 | 158.01 | 65.02 | 12.79 | 133.89 | 4.54 | 4.43 | 43.18 |
|  |  |  | BA | BA14 | SO | 4 | 18.57 | 163.01 | 68.39 | 12.78 | 131.95 | 4.35 | 4.16 | 40.49 |
|  |  |  | BA | BA16 | OP | 5 | 18.01 | 158.01 | 65.02 | 12.79 | 133.89 | 4.54 | 4.43 | 43.18 |
|  |  |  | PO | PO11 | SO | 4 | 18.01 | 158.01 | 65.02 | 12.79 | 133.89 | 4.54 | 4.43 | 43.18 |
|  |  |  | PO | PO23 | SO | 5 | 18.01 | 158.01 | 65.02 | 12.79 | 133.89 | 4.54 | 4.43 | 43.18 |
|  |  |  | PO | PO24 | SO | 1 | 18.01 | 158.01 | 65.02 | 12.79 | 133.89 | 4.54 | 4.43 | 43.18 |
|  |  |  | TE | TE05 | SC | 2 | 18.01 | 158.01 | 65.02 | 12.79 | 133.89 | 4.54 | 4.43 | 43.18 |
|  |  |  | TE | TE10 | SC | 1 | 18.01 | 158.01 | 65.02 | 12.79 | 133.89 | 4.54 | 4.43 | 43.18 |
|  |  |  | TE | TE15 | SC | 1 | 18.01 | 158.01 | 65.02 | 12.79 | 133.89 | 4.54 | 4.43 | 43.18 |
|  |  |  | TE | TE17 | SO | 2 | 18.01 | 158.01 | 65.02 | 12.79 | 133.89 | 4.54 | 4.43 | 43.18 |
|  |  |  | TE | TE21 | OP | 2 | 18.01 | 158.01 | 65.02 | 12.79 | 133.89 | 4.54 | 4.43 | 43.18 |
|  |  |  | TE | TE23 | SO | 3 | 18.01 | 158.01 | 65.02 | 12.79 | 133.89 | 4.54 | 4.43 | 43.18 |
|  |  |  | TE | TE24 | SO | 4 | 18.01 | 158.01 | 65.02 | 12.79 | 133.89 | 4.54 | 4.43 | 43.18 |
|  |  |  | TR | TR13 | OP | 4 | 18.01 | 158.01 | 65.02 | 12.79 | 133.89 | 4.54 | 4.43 | 43.18 |
|  |  |  | TR | TR18 | SO | 1 | 20.66 | 142.04 | 64.18 | 9.07 | 132.48 | 4.53 | 4.46 | 38.70 |
|  |  |  | TR | TR20 | SO | 3 | 18.01 | 158.01 | 65.02 | 12.79 | 133.89 | 4.54 | 4.43 | 43.18 |
|  |  |  | VE | VE19 | SC | 2 | 16.13 | 161.00 | 62.07 | 14.66 | 136.53 | 4.74 | 4.68 | 48.10 |
|  |  | *Leptotila rufaxilla* | BA | BA12 | SC | 2 | 27.37 | 240.33 | 89.20 | 22.76 | 187.33 | 6.21 | 5.08 | 119.27 |
|  |  |  | BA | BA16 | OP | 2 | 27.37 | 240.33 | 89.20 | 22.76 | 187.33 | 6.21 | 5.08 | 119.27 |
|  |  |  | ES | ES06 | SC | 1 | 27.60 | 240.00 | 88.90 | 22.30 | 187.00 | 5.98 | 5.01 | 118.90 |
|  |  |  | ES | ES07 | SC | 1 | 26.50 | 235.00 | 86.70 | 21.98 | 185.00 | 5.76 | 4.99 | 119.20 |
|  |  |  | PO | PO09 | SO | 2 | 27.37 | 240.33 | 89.20 | 22.76 | 187.33 | 6.21 | 5.08 | 119.27 |
|  |  |  | PO | PO11 | SO | 2 | 27.37 | 240.33 | 89.20 | 22.76 | 187.33 | 6.21 | 5.08 | 119.27 |
|  |  |  | PO | PO23 | SO | 2 | 27.37 | 240.33 | 89.20 | 22.76 | 187.33 | 6.21 | 5.08 | 119.27 |
|  |  |  | TR | TR11 | SO | 1 | 28.00 | 246.00 | 92.00 | 24.00 | 190.00 | 6.88 | 5.23 | 119.70 |
|  |  |  | TR | TR16 | OP | 1 | 27.37 | 240.33 | 89.20 | 22.76 | 187.33 | 6.21 | 5.08 | 119.27 |
|  |  | *Patagioenas cayennensis* | BA | BA01 | SO | 2 | 24.50 | 245.00 | 112.70 | 23.40 | 258.00 | 4.60 | 5.50 | 229.00 |
|  |  |  | BA | BA03 | OP | 12 | 24.50 | 245.00 | 112.70 | 23.40 | 258.00 | 4.60 | 5.50 | 229.00 |
|  |  |  | BA | BA07 | SO | 2 | 24.50 | 245.00 | 112.70 | 23.40 | 258.00 | 4.60 | 5.50 | 229.00 |
|  |  |  | BA | BA08 | SO | 2 | 24.50 | 245.00 | 112.70 | 23.40 | 258.00 | 4.60 | 5.50 | 229.00 |
|  |  |  | BA | BA09 | SO | 1 | 24.50 | 245.00 | 112.70 | 23.40 | 258.00 | 4.60 | 5.50 | 229.00 |
|  |  |  | BA | BA14 | SO | 2 | 24.50 | 245.00 | 112.70 | 23.40 | 258.00 | 4.60 | 5.50 | 229.00 |
|  |  |  | BA | BA15 | OP | 1 | 24.50 | 245.00 | 112.70 | 23.40 | 258.00 | 4.60 | 5.50 | 229.00 |
|  |  |  | BA | BA17 | SO | 2 | 24.50 | 245.00 | 112.70 | 23.40 | 258.00 | 4.60 | 5.50 | 229.00 |
|  |  |  | BA | BA18 | SC | 4 | 24.50 | 245.00 | 112.70 | 23.40 | 258.00 | 4.60 | 5.50 | 229.00 |
|  |  |  | BA | BA19 | SC | 2 | 24.50 | 245.00 | 112.70 | 23.40 | 258.00 | 4.60 | 5.50 | 229.00 |
|  |  |  | PO | PO03 | SO | 2 | 24.50 | 245.00 | 112.70 | 23.40 | 258.00 | 4.60 | 5.50 | 229.00 |
|  |  |  | PO | PO04 | SC | 1 | 24.50 | 245.00 | 112.70 | 23.40 | 258.00 | 4.60 | 5.50 | 229.00 |
|  |  |  | PO | PO05 | SC | 2 | 24.50 | 245.00 | 112.70 | 23.40 | 258.00 | 4.60 | 5.50 | 229.00 |
|  |  |  | PO | PO06 | SC | 1 | 24.50 | 245.00 | 112.70 | 23.40 | 258.00 | 4.60 | 5.50 | 229.00 |
|  |  |  | PO | PO07 | SO | 1 | 24.50 | 245.00 | 112.70 | 23.40 | 258.00 | 4.60 | 5.50 | 229.00 |
|  |  |  | PO | PO09 | SO | 3 | 24.50 | 245.00 | 112.70 | 23.40 | 258.00 | 4.60 | 5.50 | 229.00 |
|  |  |  | PO | PO11 | SA | 2 | 24.50 | 245.00 | 112.70 | 23.40 | 258.00 | 4.60 | 5.50 | 229.00 |
|  |  |  | PO | PO13 | OP | 3 | 24.50 | 245.00 | 112.70 | 23.40 | 258.00 | 4.60 | 5.50 | 229.00 |
|  |  |  | PO | PO14 | OP | 1 | 24.50 | 245.00 | 112.70 | 23.40 | 258.00 | 4.60 | 5.50 | 229.00 |
|  |  |  | PO | PO15 | SO | 2 | 24.50 | 245.00 | 112.70 | 23.40 | 258.00 | 4.60 | 5.50 | 229.00 |
|  |  |  | PO | PO17 | OP | 2 | 24.50 | 245.00 | 112.70 | 23.40 | 258.00 | 4.60 | 5.50 | 229.00 |
|  |  |  | PO | PO19 | OP | 1 | 24.50 | 245.00 | 112.70 | 23.40 | 258.00 | 4.60 | 5.50 | 229.00 |
|  |  |  | PO | PO23 | SO | 6 | 24.50 | 245.00 | 112.70 | 23.40 | 258.00 | 4.60 | 5.50 | 229.00 |
|  |  |  | PO | PO24 | SO | 4 | 24.50 | 245.00 | 112.70 | 23.40 | 258.00 | 4.60 | 5.50 | 229.00 |
|  |  |  | PO | PO25 | SO | 1 | 24.50 | 245.00 | 112.70 | 23.40 | 258.00 | 4.60 | 5.50 | 229.00 |
|  |  |  | SR | SR18 | SC | 1 | 24.50 | 245.00 | 112.70 | 23.40 | 258.00 | 4.60 | 5.50 | 229.00 |
|  |  |  | SR | SR21 | SC | 1 | 24.50 | 245.00 | 112.70 | 23.40 | 258.00 | 4.60 | 5.50 | 229.00 |
|  |  |  | TE | TE04 | SC | 4 | 24.50 | 245.00 | 112.70 | 23.40 | 258.00 | 4.60 | 5.50 | 229.00 |
|  |  |  | TE | TE17 | SO | 1 | 24.50 | 245.00 | 112.70 | 23.40 | 258.00 | 4.60 | 5.50 | 229.00 |
|  |  |  | TE | TE23 | SO | 2 | 24.50 | 245.00 | 112.70 | 23.40 | 258.00 | 4.60 | 5.50 | 229.00 |
|  |  |  | TR | TR04 | SO | 1 | 24.50 | 245.00 | 112.70 | 23.40 | 258.00 | 4.60 | 5.50 | 229.00 |
|  |  |  | TR | TR09 | OP | 2 | 24.50 | 245.00 | 112.70 | 23.40 | 258.00 | 4.60 | 5.50 | 229.00 |
|  |  |  | TR | TR18 | SO | 3 | 24.50 | 245.00 | 112.70 | 23.40 | 258.00 | 4.60 | 5.50 | 229.00 |
|  |  |  | TR | TR19 | SO | 4 | 24.50 | 245.00 | 112.70 | 23.40 | 258.00 | 4.60 | 5.50 | 229.00 |
|  |  |  | TR | TR20 | SO | 3 | 24.50 | 245.00 | 112.70 | 23.40 | 258.00 | 4.60 | 5.50 | 229.00 |
|  |  |  | VE | VE01 | OP | 1 | 24.50 | 245.00 | 112.70 | 23.40 | 258.00 | 4.60 | 5.50 | 229.00 |
|  |  |  | VE | VE14 | SC | 1 | 24.50 | 245.00 | 112.70 | 23.40 | 258.00 | 4.60 | 5.50 | 229.00 |
|  |  |  | VE | VE15 | SC | 2 | 24.50 | 245.00 | 112.70 | 23.40 | 258.00 | 4.60 | 5.50 | 229.00 |
|  |  |  | VM | VM02 | SO | 1 | 24.50 | 245.00 | 112.70 | 23.40 | 258.00 | 4.60 | 5.50 | 229.00 |
|  |  |  | VM | VM03 | SO | 1 | 24.50 | 245.00 | 112.70 | 23.40 | 258.00 | 4.60 | 5.50 | 229.00 |
|  |  |  | VM | VM05 | SO | 1 | 24.50 | 245.00 | 112.70 | 23.40 | 258.00 | 4.60 | 5.50 | 229.00 |
|  |  |  | VM | VM07 | SO | 2 | 24.50 | 245.00 | 112.70 | 23.40 | 258.00 | 4.60 | 5.50 | 229.00 |
|  |  |  | VM | VM18 | OP | 1 | 24.50 | 245.00 | 112.70 | 23.40 | 258.00 | 4.60 | 5.50 | 229.00 |
|  |  | *Patagioenas plumbea* | BA | BA12 | SC | 1 | 23.10 | 340.00 | 141.60 | 23.10 | 316.47 | 4.30 | 5.10 | 178.80 |
|  |  |  | PO | PO03 | SO | 1 | 23.10 | 340.00 | 141.60 | 23.10 | 316.47 | 4.30 | 5.10 | 178.80 |
|  |  |  | TE | TE02 | SC | 1 | 23.10 | 340.00 | 141.60 | 23.10 | 316.47 | 4.30 | 5.10 | 178.80 |
|  |  | *Patagioenas subvinacea* | BA | BA18 | SC | 1 | 15.90 | 297.50 | 120.70 | 22.40 | 281.14 | 3.80 | 4.10 | 167.25 |
|  |  |  | PO | PO11 | SO | 1 | 15.90 | 297.50 | 120.70 | 22.40 | 281.14 | 3.80 | 4.10 | 167.25 |
|  |  |  | PO | PO19 | OP | 2 | 15.90 | 297.50 | 120.70 | 22.40 | 281.14 | 3.80 | 4.10 | 167.25 |
|  |  |  | VM | VM04 | OP | 2 | 15.90 | 297.50 | 120.70 | 22.40 | 281.14 | 3.80 | 4.10 | 167.25 |
|  |  | *Zenaida auriculata** | TR | TR17 | OP | 2 | 17.50 | 250.00 | 78.95 | 19.00 | 200.00 | 3.70 | 4.00 | 110.10 |
| Passeriformes | Passerellidae | *Ammodramus aurifrons* | BA | BA12 | SC | 2 | 15.43 | 128.14 | 43.13 | 18.03 | 86.11 | 6.54 | 6.21 | 18.30 |
|  |  |  | BA | BA18 | SC | 8 | 15.43 | 128.14 | 43.13 | 18.03 | 86.11 | 6.54 | 6.21 | 18.30 |
|  |  |  | ES | ES02 | SC | 2 | 15.43 | 128.14 | 43.13 | 18.03 | 86.11 | 6.54 | 6.21 | 18.30 |
|  |  |  | ES | ES12 | SC | 2 | 13.85 | 120.25 | 41.65 | 25.63 | 59.36 | 5.66 | 5.21 | 17.93 |
|  |  |  | ES | ES14 | SC | 3 | 15.43 | 128.14 | 43.13 | 18.03 | 86.11 | 6.54 | 6.21 | 18.30 |
|  |  |  | PO | PO13 | OP | 4 | 16.09 | 128.73 | 43.92 | 17.32 | 87.21 | 6.87 | 6.41 | 18.48 |
|  |  |  | PO | PO14 | OP | 4 | 15.40 | 128.57 | 42.51 | 16.46 | 89.56 | 6.21 | 5.69 | 18.15 |
|  |  |  | PO | PO17 | OP | 1 | 15.43 | 128.14 | 43.13 | 18.03 | 86.11 | 6.54 | 6.21 | 18.30 |
|  |  |  | PO | PO18 | OP | 3 | 15.43 | 128.14 | 43.13 | 18.03 | 86.11 | 6.54 | 6.21 | 18.30 |
|  |  |  | PO | PO22 | SC | 1 | 15.44 | 130.30 | 40.28 | 17.37 | 98.30 | 6.70 | 6.16 | 19.70 |
|  |  |  | PO | PO23 | SO | 1 | 15.43 | 128.14 | 43.13 | 18.03 | 86.11 | 6.54 | 6.21 | 18.30 |
|  |  |  | PO | PO24 | SO | 2 | 15.43 | 128.14 | 43.13 | 18.03 | 86.11 | 6.54 | 6.21 | 18.30 |
|  |  |  | SR | SR06 | SO | 2 | 15.43 | 128.14 | 43.13 | 18.03 | 86.11 | 6.54 | 6.21 | 18.30 |
|  |  |  | TE | TE05 | SC | 3 | 15.43 | 128.14 | 43.13 | 18.03 | 86.11 | 6.54 | 6.21 | 18.30 |
|  |  |  | TE | TE06 | SC | 2 | 15.43 | 128.14 | 43.13 | 18.03 | 86.11 | 6.54 | 6.21 | 18.30 |
|  |  |  | TR | TR01 | OP | 2 | 15.43 | 128.14 | 43.13 | 18.03 | 86.11 | 6.54 | 6.21 | 18.30 |
|  |  |  | TR | TR02 | OP | 2 | 15.43 | 128.14 | 43.13 | 18.03 | 86.11 | 6.54 | 6.21 | 18.30 |
|  |  |  | TR | TR03 | OP | 2 | 15.43 | 128.14 | 43.13 | 18.03 | 86.11 | 6.54 | 6.21 | 18.30 |
|  |  |  | TR | TR06 | SO | 2 | 15.43 | 128.14 | 43.13 | 18.03 | 86.11 | 6.54 | 6.21 | 18.30 |
|  |  |  | TR | TR07 | SO | 2 | 15.43 | 128.14 | 43.13 | 18.03 | 86.11 | 6.54 | 6.21 | 18.30 |
|  |  |  | TR | TR08 | OP | 2 | 15.43 | 128.14 | 43.13 | 18.03 | 86.11 | 6.54 | 6.21 | 18.30 |
|  |  |  | TR | TR09 | OP | 3 | 15.43 | 128.14 | 43.13 | 18.03 | 86.11 | 6.54 | 6.21 | 18.30 |
|  |  |  | TR | TR10 | OP | 2 | 15.43 | 128.14 | 43.13 | 18.03 | 86.11 | 6.54 | 6.21 | 18.30 |
|  |  |  | TR | TR13 | OP | 2 | 15.43 | 128.14 | 43.13 | 18.03 | 86.11 | 6.54 | 6.21 | 18.30 |
|  |  |  | TR | TR14 | OP | 3 | 15.43 | 128.14 | 43.13 | 18.03 | 86.11 | 6.54 | 6.21 | 18.30 |
|  |  |  | TR | TR18 | SO | 1 | 22.24 | 127.65 | 47.98 | 18.93 | 90.95 | 11.72 | 11.09 | 19.10 |
|  |  |  | TR | TR20 | SO | 3 | 15.43 | 128.14 | 43.13 | 18.03 | 86.11 | 6.54 | 6.21 | 18.30 |
|  |  |  | TR | TR22 | OP | 1 | 15.43 | 128.14 | 43.13 | 18.03 | 86.11 | 6.54 | 6.21 | 18.30 |
|  |  |  | TR | TR24 | OP | 2 | 15.43 | 128.14 | 43.13 | 18.03 | 86.11 | 6.54 | 6.21 | 18.30 |
|  |  |  | TR | TR25 | OP | 2 | 15.43 | 128.14 | 43.13 | 18.03 | 86.11 | 6.54 | 6.21 | 18.30 |
|  |  |  | VE | VE01 | OP | 1 | 15.43 | 128.14 | 43.13 | 18.03 | 86.11 | 6.54 | 6.21 | 18.30 |
|  |  |  | VE | VE02 | OP | 3 | 15.58 | 131.84 | 43.58 | 17.51 | 89.42 | 6.53 | 5.93 | 17.65 |
|  |  |  | VM | VM01 | SO | 2 | 15.43 | 128.14 | 43.13 | 18.03 | 86.11 | 6.54 | 6.21 | 18.30 |
|  |  |  | VM | VM02 | SO | 2 | 15.43 | 128.14 | 43.13 | 18.03 | 86.11 | 6.54 | 6.21 | 18.30 |
|  |  |  | VM | VM03 | SO | 3 | 15.43 | 128.14 | 43.13 | 18.03 | 86.11 | 6.54 | 6.21 | 18.30 |
|  |  |  | VM | VM04 | OP | 3 | 14.00 | 128.72 | 42.99 | 16.87 | 88.71 | 5.75 | 6.00 | 18.24 |
|  |  |  | VM | VM05 | SO | 1 | 15.43 | 128.14 | 43.13 | 18.03 | 86.11 | 6.54 | 6.21 | 18.30 |
|  |  |  | VM | VM17 | OP | 1 | 13.28 | 129.00 | 42.91 | 16.28 | 90.00 | 5.34 | 5.88 | 18.20 |
|  |  |  | VM | VM19 | OP | 2 | 15.43 | 128.14 | 43.13 | 18.03 | 86.11 | 6.54 | 6.21 | 18.30 |
|  |  | *Arremonops conirostris* | BA | BA07 | SO | 2 | 18.79 | 166.19 | 64.17 | 19.09 | 117.09 | 8.66 | 8.68 | 35.21 |
|  |  |  | BA | BA08 | SO | 1 | 18.79 | 166.19 | 64.17 | 19.09 | 117.09 | 8.66 | 8.68 | 35.21 |
|  |  |  | BA | BA09 | SO | 1 | 18.79 | 166.19 | 64.17 | 19.09 | 117.09 | 8.66 | 8.68 | 35.21 |
|  |  |  | BA | BA10 | SC | 2 | 18.79 | 166.19 | 64.17 | 19.09 | 117.09 | 8.66 | 8.68 | 35.21 |
|  |  |  | BA | BA12 | SC | 3 | 18.79 | 166.19 | 64.17 | 19.09 | 117.09 | 8.66 | 8.68 | 35.21 |
|  |  |  | BA | BA13 | SC | 1 | 18.79 | 166.19 | 64.17 | 19.09 | 117.09 | 8.66 | 8.68 | 35.21 |
|  |  |  | BA | BA16 | OP | 4 | 18.79 | 166.19 | 64.17 | 19.09 | 117.09 | 8.66 | 8.68 | 35.21 |
|  |  |  | BA | BA18 | SC | 2 | 18.79 | 166.19 | 64.17 | 19.09 | 117.09 | 8.66 | 8.68 | 35.21 |
|  |  |  | PO | PO05 | SC | 2 | 18.79 | 166.19 | 64.17 | 19.09 | 117.09 | 8.66 | 8.68 | 35.21 |
|  |  |  | PO | PO14 | OP | 3 | 18.05 | 164.73 | 60.88 | 18.72 | 112.71 | 7.92 | 8.61 | 35.37 |
|  |  |  | PO | PO15 | SO | 1 | 18.79 | 166.19 | 64.17 | 19.09 | 117.09 | 8.66 | 8.68 | 35.21 |
|  |  |  | PO | PO18 | OP | 1 | 18.79 | 166.19 | 64.17 | 19.09 | 117.09 | 8.66 | 8.68 | 35.21 |
|  |  |  | PO | PO23 | SO | 6 | 18.79 | 166.19 | 64.17 | 19.09 | 117.09 | 8.66 | 8.68 | 35.21 |
|  |  |  | TE | TE05 | SC | 2 | 18.79 | 166.19 | 64.17 | 19.09 | 117.09 | 8.66 | 8.68 | 35.21 |
|  |  |  | TE | TE08 | SC | 2 | 18.79 | 166.19 | 64.17 | 19.09 | 117.09 | 8.66 | 8.68 | 35.21 |
|  |  |  | TE | TE14 | SC | 2 | 20.18 | 180.60 | 70.24 | 15.98 | 128.62 | 11.62 | 7.36 | 34.35 |
|  |  |  | TE | TE17 | SO | 2 | 18.79 | 166.19 | 64.17 | 19.09 | 117.09 | 8.66 | 8.68 | 35.21 |
|  |  |  | TR | TR01 | OP | 2 | 18.79 | 166.19 | 64.17 | 19.09 | 117.09 | 8.66 | 8.68 | 35.21 |
|  |  |  | TR | TR09 | OP | 2 | 18.79 | 166.19 | 64.17 | 19.09 | 117.09 | 8.66 | 8.68 | 35.21 |
|  |  |  | TR | TR15 | OP | 2 | 18.46 | 136.15 | 63.41 | 16.51 | 112.56 | 7.86 | 7.75 | 33.36 |
|  |  |  | TR | TR18 | SO | 4 | 20.54 | 167.63 | 62.50 | 20.79 | 112.30 | 9.25 | 9.82 | 36.11 |
|  |  |  | TR | TR20 | SO | 4 | 18.79 | 166.19 | 64.17 | 19.09 | 117.09 | 8.66 | 8.68 | 35.21 |
|  |  |  | TR | TR22 | OP | 2 | 18.79 | 166.19 | 64.17 | 19.09 | 117.09 | 8.66 | 8.68 | 35.21 |
|  |  |  | VM | VM07 | SO | 1 | 17.82 | 172.00 | 63.00 | 21.58 | 124.00 | 7.34 | 8.40 | 36.10 |
|  |  |  | VM | VM08 | SO | 1 | 18.52 | 172.70 | 63.70 | 22.28 | 124.70 | 8.04 | 9.10 | 36.80 |
|  |  |  | VM | VM17 | OP | 1 | 15.99 | 175.00 | 68.00 | 19.14 | 119.00 | 7.45 | 8.75 | 34.70 |
|  |  |  | VM | VM18 | OP | 1 | 15.94 | 174.95 | 67.95 | 19.09 | 118.95 | 7.40 | 8.70 | 34.65 |
|  | Thraupidae | *Sicalis flaveola* | BA | BA04 | OP | 2 | 16.51 | 145.00 | 54.41 | 13.53 | 123.51 | 6.07 | 7.71 | 21.80 |
|  |  |  | BA | BA05 | OP | 2 | 16.51 | 145.00 | 54.41 | 13.53 | 123.51 | 6.07 | 7.71 | 21.80 |
|  |  |  | BA | BA07 | SO | 1 | 16.51 | 145.00 | 54.41 | 13.53 | 123.51 | 6.07 | 7.71 | 21.80 |
|  |  |  | BA | BA09 | SO | 1 | 16.51 | 145.00 | 54.41 | 13.53 | 123.51 | 6.07 | 7.71 | 21.80 |
|  |  |  | BA | BA10 | SC | 2 | 16.51 | 145.00 | 54.41 | 13.53 | 123.51 | 6.07 | 7.71 | 21.80 |
|  |  |  | BA | BA12 | SC | 1 | 16.51 | 145.00 | 54.41 | 13.53 | 123.51 | 6.07 | 7.71 | 21.80 |
|  |  |  | BA | BA14 | SO | 2 | 16.51 | 145.00 | 54.41 | 13.53 | 123.51 | 6.07 | 7.71 | 21.80 |
|  |  |  | BA | BA15 | OP | 2 | 16.51 | 145.00 | 54.41 | 13.53 | 123.51 | 6.07 | 7.71 | 21.80 |
|  |  |  | BA | BA17 | SO | 6 | 16.51 | 145.00 | 54.41 | 13.53 | 123.51 | 6.07 | 7.71 | 21.80 |
|  |  |  | BA | BA18 | SC | 3 | 16.51 | 145.00 | 54.41 | 13.53 | 123.51 | 6.07 | 7.71 | 21.80 |
|  |  |  | PO | PO07 | SO | 2 | 16.51 | 145.00 | 54.41 | 13.53 | 123.51 | 6.07 | 7.71 | 21.80 |
|  |  |  | PO | PO13 | OP | 2 | 16.51 | 145.00 | 54.41 | 13.53 | 123.51 | 6.07 | 7.71 | 21.80 |
|  |  |  | PO | PO14 | OP | 2 | 16.51 | 145.00 | 54.41 | 13.53 | 123.51 | 6.07 | 7.71 | 21.80 |
|  |  |  | PO | PO15 | SO | 2 | 16.51 | 145.00 | 54.41 | 13.53 | 123.51 | 6.07 | 7.71 | 21.80 |
|  |  |  | PO | PO18 | OP | 3 | 16.51 | 145.00 | 54.41 | 13.53 | 123.51 | 6.07 | 7.71 | 21.80 |
|  |  |  | PO | PO22 | SC | 3 | 16.51 | 145.00 | 54.41 | 13.53 | 123.51 | 6.07 | 7.71 | 21.80 |
|  |  |  | PO | PO23 | SO | 2 | 16.51 | 145.00 | 54.41 | 13.53 | 123.51 | 6.07 | 7.71 | 21.80 |
|  |  |  | PO | PO24 | SO | 3 | 16.51 | 145.00 | 54.41 | 13.53 | 123.51 | 6.07 | 7.71 | 21.80 |
|  |  |  | PO | PO25 | SO | 2 | 16.51 | 145.00 | 54.41 | 13.53 | 123.51 | 6.07 | 7.71 | 21.80 |
|  |  |  | TE | TE01 | SC | 3 | 16.51 | 145.00 | 54.41 | 13.53 | 123.51 | 6.07 | 7.71 | 21.80 |
|  |  |  | TE | TE06 | SC | 2 | 16.51 | 145.00 | 54.41 | 13.53 | 123.51 | 6.07 | 7.71 | 21.80 |
|  |  |  | TR | TR21 | OP | 2 | 16.51 | 145.00 | 54.41 | 13.53 | 123.51 | 6.07 | 7.71 | 21.80 |
|  |  |  | VE | VE02 | OP | 2 | 16.51 | 145.00 | 54.41 | 13.53 | 123.51 | 6.07 | 7.71 | 21.80 |
|  |  |  | VE | VE03 | SO | 2 | 16.51 | 145.00 | 54.41 | 13.53 | 123.51 | 6.07 | 7.71 | 21.80 |
|  |  |  | VM | VM04 | OP | 3 | 16.51 | 145.00 | 54.41 | 13.53 | 123.51 | 6.07 | 7.71 | 21.80 |
|  |  |  | VM | VM18 | OP | 2 | 16.51 | 145.00 | 54.41 | 13.53 | 123.51 | 6.07 | 7.71 | 21.80 |
|  |  |  | VM | VM19 | OP | 2 | 16.51 | 145.00 | 54.41 | 13.53 | 123.51 | 6.07 | 7.71 | 21.80 |
|  |  | *Sporophila angolensis* | BA | BA07 | SO | 3 | 13.00 | 123.00 | 47.00 | 11.67 | 86.33 | 7.83 | 8.94 | 12.03 |
|  |  |  | BA | BA08 | SO | 1 | 12.89 | 115.22 | 44.98 | 11.85 | 89.13 | 7.37 | 9.10 | 12.10 |
|  |  |  | BA | BA14 | SO | 1 | 13.75 | 116.00 | 53.29 | 11.83 | 79.48 | 6.78 | 9.82 | 12.03 |
|  |  |  | BA | BA17 | SO | 2 | 13.29 | 120.90 | 45.54 | 12.64 | 78.81 | 7.42 | 8.66 | 11.52 |
|  |  |  | BA | BA18 | SC | 2 | 13.29 | 120.90 | 45.54 | 12.64 | 78.81 | 7.42 | 8.66 | 11.52 |
|  |  |  | ES | ES03 | SC | 2 | 8.96 | 119.97 | 38.51 | 12.40 | 64.41 | 8.21 | 9.62 | 10.10 |
|  |  |  | ES | ES06 | SC | 1 | 24.93 | 115.30 | 11.52 | 34.78 | 22.76 | 3.26 | 2.25 | 4.70 |
|  |  |  | PO | PO03 | SO | 2 | 13.29 | 120.90 | 45.54 | 12.64 | 78.81 | 7.42 | 8.66 | 11.52 |
|  |  |  | PO | PO11 | SO | 2 | 13.29 | 120.90 | 45.54 | 12.64 | 78.81 | 7.42 | 8.66 | 11.52 |
|  |  |  | PO | PO18 | OP | 2 | 13.29 | 120.90 | 45.54 | 12.64 | 78.81 | 7.42 | 8.66 | 11.52 |
|  |  |  | PO | PO24 | SO | 2 | 13.29 | 120.90 | 45.54 | 12.64 | 78.81 | 7.42 | 8.66 | 11.52 |
|  |  |  | TE | TE06 | SC | 1 | 13.29 | 120.90 | 45.54 | 12.64 | 78.81 | 7.42 | 8.66 | 11.52 |
|  |  |  | TE | TE14 | SC | 2 | 13.19 | 119.63 | 46.72 | 9.10 | 87.34 | 7.76 | 8.60 | 12.65 |
|  |  |  | TR | TR18 | SO | 3 | 14.16 | 116.64 | 50.13 | 12.20 | 81.02 | 7.62 | 8.94 | 10.61 |
|  |  |  | TR | TR19 | SO | 2 | 12.29 | 118.18 | 51.26 | 10.72 | 84.16 | 7.90 | 8.99 | 10.40 |
|  |  |  | VE | VE02 | OP | 1 | 13.14 | 125.94 | 54.14 | 10.28 | 89.39 | 7.49 | 9.36 | 12.40 |
|  |  |  | VM | VM07 | SO | 1 | 12.76 | 128.00 | 54.05 | 11.26 | 86.00 | 7.96 | 9.27 | 11.80 |
|  |  |  | VM | VM18 | OP | 1 | 12.21 | 137.00 | 42.00 | 10.98 | 80.20 | 6.90 | 8.35 | 19.20 |
|  |  | *Sporophila castaneiventris* | TE | TE06 | SC | 2 | 11.00 | 100.00 | 37.50 | 13.80 | 91.33 | 4.60 | 5.70 | 7.80 |
|  |  |  | TE | TE21 | OP | 2 | 11.00 | 100.00 | 37.50 | 13.80 | 91.33 | 4.60 | 5.70 | 7.80 |
|  |  |  | VM | VM01 | SO | 2 | 11.00 | 100.00 | 37.50 | 13.80 | 91.33 | 4.60 | 5.70 | 7.80 |
|  |  | *Sporophila crassirostris* | VE | VE02 | OP | 2 | 13.56 | 122.38 | 49.90 | 10.86 | 89.79 | 7.60 | 8.74 | 11.60 |
|  |  |  | VE | VE19 | SC | 1 | 12.87 | 111.19 | 41.39 | 10.93 | 75.55 | 8.67 | 8.29 | 11.00 |
|  |  | *Sporophila intermedia*** | TR | TR18 | SO | 1 | 12.31 | 104.21 | 37.19 | 5.57 | 76.55 | 8.04 | 8.89 | 12.20 |
|  |  | *Sporophila minuta*** | TR | TR18 | SO | 1 | 9.15 | 90.00 | 38.99 | 14.15 | 86.85 | 5.00 | 6.20 | 7.90 |
|  |  | *Sporophila murallae* | BA | BA07 | SO | 4 | 26.38 | 125.00 | 48.48 | 11.37 | 90.50 | 7.63 | 8.11 | 14.75 |
|  |  |  | BA | BA13 | SC | 2 | 26.38 | 125.00 | 48.48 | 11.37 | 90.50 | 7.63 | 8.11 | 14.75 |
|  |  |  | TR | TR18 | SO | 2 | 26.38 | 125.00 | 48.48 | 11.37 | 90.50 | 7.63 | 8.11 | 14.75 |
|  |  | *Sporophila nigricollis*** | PO | PO23 | SO | 2 | 9.39 | 111.00 | 45.00 | 20.00 | 94.30 | 6.04 | 5.84 | 9.80 |
|  |  | *Volatinia jacarina* | BA | BA07 | SO | 3 | 10.95 | 107.50 | 39.79 | 10.57 | 72.10 | 6.19 | 6.33 | 9.60 |
|  |  |  | BA | BA10 | SC | 1 | 13.08 | 102.24 | 39.12 | 11.01 | 74.31 | 9.20 | 6.84 | 9.20 |
|  |  |  | BA | BA12 | SC | 1 | 13.08 | 102.24 | 39.12 | 11.01 | 74.31 | 9.20 | 6.84 | 9.20 |
|  |  |  | BA | BA14 | SO | 2 | 13.08 | 102.24 | 39.12 | 11.01 | 74.31 | 9.20 | 6.84 | 9.20 |
|  |  |  | BA | BA16 | OP | 2 | 13.08 | 102.24 | 39.12 | 11.01 | 74.31 | 9.20 | 6.84 | 9.20 |
|  |  |  | BA | BA17 | SO | 8 | 13.08 | 102.24 | 39.12 | 11.01 | 74.31 | 9.20 | 6.84 | 9.20 |
|  |  |  | ES | ES07 | SC | 1 | 13.08 | 102.24 | 39.12 | 11.01 | 74.31 | 9.20 | 6.84 | 9.20 |
|  |  |  | ES | ES14 | SC | 2 | 13.08 | 102.24 | 39.12 | 11.01 | 74.31 | 9.20 | 6.84 | 9.20 |
|  |  |  | PO | PO03 | SO | 2 | 13.08 | 102.24 | 39.12 | 11.01 | 74.31 | 9.20 | 6.84 | 9.20 |
|  |  |  | PO | PO09 | SO | 2 | 13.08 | 102.24 | 39.12 | 11.01 | 74.31 | 9.20 | 6.84 | 9.20 |
|  |  |  | PO | PO11 | SO | 4 | 13.08 | 102.24 | 39.12 | 11.01 | 74.31 | 9.20 | 6.84 | 9.20 |
|  |  |  | PO | PO14 | OP | 2 | 13.08 | 102.24 | 39.12 | 11.01 | 74.31 | 9.20 | 6.84 | 9.20 |
|  |  |  | PO | PO15 | SO | 2 | 13.08 | 102.24 | 39.12 | 11.01 | 74.31 | 9.20 | 6.84 | 9.20 |
|  |  |  | PO | PO16 | SC | 2 | 13.08 | 102.24 | 39.12 | 11.01 | 74.31 | 9.20 | 6.84 | 9.20 |
|  |  |  | PO | PO17 | OP | 1 | 13.08 | 102.24 | 39.12 | 11.01 | 74.31 | 9.20 | 6.84 | 9.20 |
|  |  |  | PO | PO19 | OP | 2 | 13.08 | 102.24 | 39.12 | 11.01 | 74.31 | 9.20 | 6.84 | 9.20 |
|  |  |  | PO | PO22 | SC | 2 | 13.08 | 102.24 | 39.12 | 11.01 | 74.31 | 9.20 | 6.84 | 9.20 |
|  |  |  | PO | PO23 | SO | 2 | 13.08 | 102.24 | 39.12 | 11.01 | 74.31 | 9.20 | 6.84 | 9.20 |
|  |  |  | PO | PO24 | SO | 4 | 13.08 | 102.24 | 39.12 | 11.01 | 74.31 | 9.20 | 6.84 | 9.20 |
|  |  |  | PO | PO25 | SO | 2 | 13.08 | 102.24 | 39.12 | 11.01 | 74.31 | 9.20 | 6.84 | 9.20 |
|  |  |  | SR | SR02 | SO | 1 | 10.72 | 103.00 | 43.69 | 10.71 | 79.46 | 6.44 | 6.76 | 10.30 |
|  |  |  | TE | TE06 | SC | 1 | 13.08 | 102.24 | 39.12 | 11.01 | 74.31 | 9.20 | 6.84 | 9.20 |
|  |  |  | TE | TE21 | OP | 1 | 13.08 | 102.24 | 39.12 | 11.01 | 74.31 | 9.20 | 6.84 | 9.20 |
|  |  |  | TR | TR02 | OP | 2 | 13.08 | 102.24 | 39.12 | 11.01 | 74.31 | 9.20 | 6.84 | 9.20 |
|  |  |  | TR | TR03 | OP | 2 | 13.08 | 102.24 | 39.12 | 11.01 | 74.31 | 9.20 | 6.84 | 9.20 |
|  |  |  | TR | TR07 | SO | 1 | 13.08 | 102.24 | 39.12 | 11.01 | 74.31 | 9.20 | 6.84 | 9.20 |
|  |  |  | TR | TR08 | OP | 2 | 13.08 | 102.24 | 39.12 | 11.01 | 74.31 | 9.20 | 6.84 | 9.20 |
|  |  |  | TR | TR09 | OP | 1 | 13.08 | 102.24 | 39.12 | 11.01 | 74.31 | 9.20 | 6.84 | 9.20 |
|  |  |  | TR | TR10 | OP | 3 | 13.08 | 102.24 | 39.12 | 11.01 | 74.31 | 9.20 | 6.84 | 9.20 |
|  |  |  | TR | TR13 | OP | 6 | 13.08 | 102.24 | 39.12 | 11.01 | 74.31 | 9.20 | 6.84 | 9.20 |
|  |  |  | TR | TR14 | OP | 2 | 13.08 | 102.24 | 39.12 | 11.01 | 74.31 | 9.20 | 6.84 | 9.20 |
|  |  |  | TR | TR18 | SO | 4 | 15.03 | 99.24 | 38.99 | 11.14 | 75.07 | 9.09 | 7.69 | 8.40 |
|  |  |  | TR | TR19 | SO | 1 | 13.81 | 89.00 | 34.10 | 10.75 | 73.22 | 6.19 | 6.58 | 9.10 |
|  |  |  | TR | TR22 | OP | 2 | 13.08 | 102.24 | 39.12 | 11.01 | 74.31 | 9.20 | 6.84 | 9.20 |
|  |  |  | TR | TR24 | OP | 2 | 13.08 | 102.24 | 39.12 | 11.01 | 74.31 | 9.20 | 6.84 | 9.20 |
|  |  |  | TR | TR25 | OP | 2 | 13.08 | 102.24 | 39.12 | 11.01 | 74.31 | 9.20 | 6.84 | 9.20 |
|  |  |  | VE | VE01 | OP | 1 | 13.08 | 102.24 | 39.12 | 11.01 | 74.31 | 9.20 | 6.84 | 9.20 |
|  |  |  | VE | VE02 | OP | 2 | 13.08 | 102.24 | 39.12 | 11.01 | 74.31 | 9.20 | 6.84 | 9.20 |
|  |  |  | VE | VE03 | SO | 2 | 13.08 | 102.24 | 39.12 | 11.01 | 74.31 | 9.20 | 6.84 | 9.20 |
|  |  |  | VM | VM01 | SO | 3 | 13.08 | 102.24 | 39.12 | 11.01 | 74.31 | 9.20 | 6.84 | 9.20 |
|  |  |  | VM | VM03 | SO | 2 | 13.08 | 102.24 | 39.12 | 11.01 | 74.31 | 9.20 | 6.84 | 9.20 |
|  |  |  | VM | VM04 | OP | 3 | 13.08 | 102.24 | 39.12 | 11.01 | 74.31 | 9.20 | 6.84 | 9.20 |
|  |  |  | VM | VM17 | OP | 1 | 13.30 | 111.00 | 38.11 | 12.41 | 73.85 | 6.18 | 5.33 | 10.20 |
|  |  |  | VM | VM18 | OP | 2 | 13.08 | 102.24 | 39.12 | 11.01 | 74.31 | 9.20 | 6.84 | 9.20 |
| Tinamiformes | Tinamidae | *Crypturellus cinereus**** | SR | SR05 | SC | 1 | 32.40 | 305.00 | 64.45 | 49.95 | 237.50 | 5.20 | 5.40 | 541.05 |
|  |  |  | SR | SR15 | SC | 2 | 32.40 | 305.00 | 64.45 | 49.95 | 237.50 | 5.20 | 5.40 | 541.05 |
|  |  |  | TE | TE08 | SC | 1 | 32.40 | 305.00 | 64.45 | 49.95 | 237.50 | 5.20 | 5.40 | 541.05 |
|  |  | *Crypturellus soui* | BA | BA12 | SC | 1 | 21.35 | 225.00 | 46.45 | 38.20 | 200.00 | 4.20 | 3.90 | 218.10 |
|  |  |  | PO | PO03 | SO | 2 | 21.35 | 225.00 | 46.45 | 38.20 | 200.00 | 4.20 | 3.90 | 218.10 |
|  |  |  | PO | PO05 | SC | 1 | 21.35 | 225.00 | 46.45 | 38.20 | 200.00 | 4.20 | 3.90 | 218.10 |
|  |  |  | PO | PO13 | OP | 1 | 21.35 | 225.00 | 46.45 | 38.20 | 200.00 | 4.20 | 3.90 | 218.10 |
|  |  |  | VE | VE14 | SC | 1 | 21.35 | 225.00 | 46.45 | 38.20 | 200.00 | 4.20 | 3.90 | 218.10 |
|  |  | *Crypturellus undulatus* | BA | BA12 | SC | 1 | 34.00 | 220.00 | 56.60 | 48.00 | 160.00 | 6.00 | 5.80 | 564.40 |
|  |  |  | PO | PO03 | SO | 1 | 34.00 | 220.00 | 56.60 | 48.00 | 160.00 | 6.00 | 5.80 | 564.40 |
|  |  |  | PO | PO12 | SO | 1 | 34.00 | 220.00 | 56.60 | 48.00 | 160.00 | 6.00 | 5.80 | 564.40 |
|  |  |  | PO | PO13 | OP | 1 | 34.00 | 220.00 | 56.60 | 48.00 | 160.00 | 6.00 | 5.80 | 564.40 |
|  |  |  | SR | SR06 | SO | 1 | 34.00 | 220.00 | 56.60 | 48.00 | 160.00 | 6.00 | 5.80 | 564.40 |
|  |  |  | TE | TE12 | SC | 1 | 34.00 | 220.00 | 56.60 | 48.00 | 160.00 | 6.00 | 5.80 | 564.40 |
|  |  |  | VE | VE23 | SC | 2 | 34.00 | 220.00 | 56.60 | 48.00 | 160.00 | 6.00 | 5.80 | 564.40 |
|  |  | *Tinamus guttatus**** | BA | BA12 | SC | 2 | 22.60 | 120.00 | 61.80 | 37.20 | 180.26 | 5.00 | 5.30 | 352.10 |
|  |  |  | BA | BA18 | SC | 1 | 22.60 | 120.00 | 61.80 | 37.20 | 180.26 | 5.00 | 5.30 | 352.10 |
|  |  |  | SR | SR15 | SC | 1 | 22.60 | 120.00 | 61.80 | 37.20 | 180.26 | 5.00 | 5.30 | 352.10 |

BA: Batalla 13; PO: El Porvenir; VE: La Vega; TE: El Tesoro; TR: El Triunfo; ES: La Esmeralda; SR: Santa Rosa; VM: Villa Mery; TC: tree cover; OP: open; SO: semi-open; SC: semi-closed; AB: abundance of individuals; CTO: total culmen; LTO: total body length; LCO: tail length; LTA: tarsus length; AEX: extended wing; COM: commissure; ALT: bill height; PES: body weight (g). Species recorded under a single type of cover: *open (species recorded, one); **semi-open (species recorded, three); ***semi-closed (species recorded, two).
