# Supplementary material for: Livestock landscapes as ecological filters: Effects of the tree cover gradient on the taxonomic and functional diversity of granivorous birds in the Colombian Amazon
Source: PLoS One. 2026 Mar 20;21(3):e0345283. doi: 10.1371/journal.pone.0345283 (PMC13004383; doi:10.1371/journal.pone.0345283)
Supplement: S4 Table — (DOCX) [file pone.0345283.s004.docx]

**S4 Table.** Matrix of morphological traits for each granivorous bird species recorded in the study

| **Orden** | **Family** | **Scientific name** | **CTO** | **LTO** | **LCO** | **LTA** | **AEX** | **COM** | **ALT** | **PES** |
| --- | --- | --- | --- | --- | --- | --- | --- | --- | --- | --- |
| Columbiformes | Columbidae | *Columbina minuta* | 16.03 | 148.08 | 55.22 | 11.75 | 122.63 | 5.99 | 3.43 | 35.47 |
|  |  | *Columbina talpacoti* | 18.08 | 157.57 | 65.00 | 12.69 | 133.85 | 4.54 | 4.43 | 43.06 |
|  |  | *Leptotila rufaxilla* | 27.37 | 240.33 | 89.20 | 22.76 | 187.33 | 6.21 | 5.08 | 119.27 |
|  |  | *Patagioenas cayennensis* | 24.50 | 245.00 | 112.70 | 23.40 | 258.00 | 4.60 | 5.50 | 229.00 |
|  |  | *Patagioenas plumbea* | 23.10 | 340.00 | 141.60 | 23.10 | 316.47 | 4.30 | 5.10 | 178.80 |
|  |  | *Patagioenas subvinacea* | 15.90 | 297.50 | 120.70 | 22.40 | 281.14 | 3.80 | 4.10 | 167.25 |
|  |  | *Zenaida auriculata* | 17.50 | 250.00 | 78.95 | 19.00 | 200.00 | 3.70 | 4.00 | 110.10 |
| Passeriformes | Passerellidae | *Ammodramus aurifrons* | 15.49 | 128.14 | 43.15 | 18.08 | 86.23 | 6.60 | 6.28 | 18.33 |
|  |  | *Arremonops conirostris* | 18.61 | 166.72 | 64.40 | 19.14 | 117.69 | 8.57 | 8.64 | 35.20 |
|  | Thraupidae | *Sicalis flaveola* | 16.51 | 145.00 | 54.41 | 13.53 | 123.51 | 6.07 | 7.71 | 21.80 |
|  |  | *Sporophila angolensis* | 13.57 | 121.18 | 45.13 | 13.09 | 77.88 | 7.28 | 8.55 | 11.59 |
|  |  | *Sporophila castaneiventris* | 11.00 | 100.00 | 37.50 | 13.80 | 91.33 | 4.60 | 5.70 | 7.80 |
|  |  | *Sporophila minuta* | 9.15 | 90.00 | 38.99 | 14.15 | 86.85 | 5.00 | 6.20 | 7.90 |
|  |  | *Sporophila murellae* | 26.38 | 125.00 | 48.48 | 11.37 | 90.50 | 7.63 | 8.11 | 14.75 |
|  |  | *Sporophila nigricollis* | 9.39 | 111.00 | 45.00 | 20.00 | 94.30 | 6.04 | 5.84 | 9.80 |
|  |  | *Volatinia jacarina* | 13.04 | 102.21 | 39.10 | 11.02 | 74.36 | 8.93 | 6.81 | 9.24 |
|  |  | *Sporophila intermedia* | 12.31 | 104.21 | 37.19 | 5.57 | 76.55 | 8.04 | 8.89 | 12.20 |
|  |  | *Sporophila crassirostris* | 13.22 | 116.79 | 45.65 | 10.90 | 82.67 | 8.14 | 8.52 | 11.30 |
| Tinamiformes | Tinamidae | *Crypturellus cinereus* | 32.40 | 305.00 | 64.45 | 49.95 | 237.50 | 5.20 | 5.40 | 541.05 |
|  |  | *Crypturellus soui* | 21.35 | 225.00 | 46.45 | 38.20 | 200.00 | 4.20 | 3.90 | 218.10 |
|  |  | *Crypturellus undulatus* | 34.00 | 220.00 | 56.60 | 48.00 | 160.00 | 6.00 | 5.80 | 564.40 |
|  |  | *Tinamus guttatus* | 22.60 | 120.00 | 61.80 | 37.20 | 180.26 | 5.00 | 5.30 | 352.10 |

CTO: total culmen; LTO: total body length; LCO: tail length; LTA: tarsus length; AEX: extended wing length; COM: commissure; ALT: bill height; All values are presented in millimeters (mm); PES: body weight (g).
