# Supplementary material for: Livestock landscapes as ecological filters: Effects of the tree cover gradient on the taxonomic and functional diversity of granivorous birds in the Colombian Amazon
Source: PLoS One. 2026 Mar 20;21(3):e0345283. doi: 10.1371/journal.pone.0345283 (PMC13004383; doi:10.1371/journal.pone.0345283)
Supplement: S5 Table — (DOCX) [file pone.0345283.s005.docx]

**S5 Table.** Matrix of granivorous bird species assigned to two functional groups (small and large) based on the dendrogram generated by hierarchical cluster analysis using the ward.D algorithm.

| **#** | **FG** | **Order** | **Familiy** | **Scientific name** | **AB** | **Morphological functional traits** | | | | | | | |
| --- | --- | --- | --- | --- | --- | --- | --- | --- | --- | --- | --- | --- | --- |
|  |  |  |  |  |  | **CTO** | **LTO** | **LCO** | **LTA** | **AEX** | **COM** | **ALT** | **PES** |
| 01 | FG_S | Passeriformes | Passerellidae | *Ammodramus aurifrons* | 89 | 15.49 | 128.14 | 43.15 | 18.08 | 86.23 | 6.60 | 6.28 | 18.33 |
| 02 |  |  |  | *Arremonops conirostris* | 57 | 18.68 | 166.75 | 64.34 | 19.20 | 117.49 | 8.60 | 8.69 | 35.23 |
| 03 |  |  | Thraupidae | *Sicalis flaveola* | 61 | 16.51 | 145.00 | 54.41 | 13.53 | 123.51 | 6.07 | 7.71 | 21.80 |
| 04 |  |  |  | *Sporophila angolensis* | 31 | 13.60 | 120.94 | 45.39 | 13.04 | 78.05 | 7.30 | 8.57 | 11.54 |
| 05 |  |  |  | *Sporophila castaneiventris* | 06 | 11.00 | 100.00 | 37.50 | 13.80 | 91.33 | 4.60 | 5.70 | 7.80 |
| 06 |  |  |  | *Sporophila crassirostris* | 03 | 13.22 | 116.79 | 45.65 | 10.90 | 82.67 | 8.14 | 8.52 | 11.30 |
| 07 |  |  |  | *Sporophila intermedia* | 01 | 12.31 | 104.21 | 37.19 | 5.57 | 76.55 | 8.04 | 8.89 | 12.20 |
| 08 |  |  |  | *Sporophila minuta* | 01 | 9.15 | 90.00 | 38.99 | 14.15 | 86.85 | 5.00 | 6.20 | 7.90 |
| 09 |  |  |  | *Sporophila murallae* | 08 | 26.38 | 125.00 | 48.48 | 11.37 | 90.50 | 7.63 | 8.11 | 14.75 |
| 10 |  |  |  | *Sporophila nigricollis* | 02 | 9.39 | 111.00 | 45.00 | 20.00 | 94.30 | 6.04 | 5.84 | 9.80 |
| 11 |  |  |  | *Volatinia jacarina* | 96 | 13.09 | 102.14 | 39.10 | 11.02 | 74.37 | 8.93 | 6.83 | 9.22 |
| 12 |  | Columbiformes | Columbidae | *Columbina minuta* | 15 | 16.03 | 148.08 | 55.22 | 11.75 | 122.63 | 5.99 | 3.43 | 35.47 |
| 13 |  |  |  | *Columbina talpacoti* | 49 | 18.08 | 157.57 | 65.00 | 12.69 | 133.85 | 4.54 | 4.43 | 43.06 |
|  |  |  |  |  | **M** | **15.21** | **128.68** | **47.63** | **14.87** | **93.75** | **7.51** | **7.31** | **18.65** |
|  |  |  |  |  | **SD** | **2.64** | **22.99** | **9.67** | **3.80** | **20.58** | **1.45** | **1.18** | **9.05** |
| 14 | FG_L | Tinamiformes | Tinamidae | *Crypturellus soui* | 04 | 32.40 | 305.00 | 64.45 | 49.95 | 237.50 | 5.20 | 5.40 | 541.05 |
| 15 |  |  |  | *Crypturellus cinereus* | 06 | 21.35 | 225.00 | 46.45 | 38.20 | 200.00 | 4.20 | 3.90 | 218.10 |
| 16 |  |  |  | *Crypturellus undulatus* | 08 | 34.00 | 220.00 | 56.60 | 48.00 | 160.00 | 6.00 | 5.80 | 564.40 |
| 17 |  |  |  | *Tinamus guttatus* | 04 | 22.60 | 220.00 | 71.80 | 37.20 | 180.26 | 5.00 | 5.30 | 352.10 |
| 18 |  | Columbiformes | Columbidae | *Leptotila rufaxilla* | 14 | 27.37 | 240.33 | 89.20 | 22.76 | 187.33 | 6.21 | 5.08 | 119.27 |
| 19 |  |  |  | *Patagioenas cayennensis* | 94 | 24.50 | 245.00 | 112.70 | 23.40 | 258.00 | 4.60 | 5.50 | 229.00 |
| 20 |  |  |  | *Patagioenas plumbea* | 03 | 23.10 | 340.00 | 141.60 | 23.10 | 316.47 | 4.30 | 5.10 | 178.80 |
| 21 |  |  |  | *Patagioenas subvinacea* | 06 | 15.90 | 297.50 | 120.70 | 22.40 | 281.14 | 3.80 | 4.10 | 167.25 |
| 22 |  |  |  | *Zenaida auriculata* | 02 | 17.50 | 250.00 | 78.95 | 19.00 | 200.00 | 3.70 | 4.00 | 110.10 |
|  |  |  |  |  | **M** | **23.28** | **223.02** | **89.31** | **24.15** | **210.63** | **4.88** | **4.99** | **203.69** |
|  |  |  |  |  | **SD** | **4.88** | **50.14** | **27.19** | **10.40** | **57.65** | **0.92** | **0.70** | **143.13** |

FG: functional group; GF_S: functional group of small granivorous birds; FG_L: functional group of large granivorous birds; AB: abundance of individuals; ALT: bill height; COM: commissure; AEX: extended wing; LTA: tarsus length; LCO: tail length; LTO: total body length; CTO: total culmen; PES: body weight (g); M: trait mean; SD: standard deviation.
